# Scavenging Reactive Oxygen Species by Cerium Oxide Nanoparticles Prevents Death in a Peripheral T Cell Lymphoma Preclinical Mouse Model

**DOI:** 10.1021/acsnano.5c02860

**Published:** 2025-05-09

**Authors:** Adrien Krug, Lena M. Ernst, Rana Mhaidly, Joana Ramis, Muriel F. Gusta, Neus G. Bastus, Adriana Martinez-Turtos, Marie Tosolini, Léa Di Mascio, Gamze Tari, Laurent Boyer, Philippe Gaulard, François Lemonnier, Jean-Ehrland Ricci, Els Verhoeyen, Victor Puntes

**Affiliations:** 1 Université Côte d’Azur, INSERM, C3M, Nice 06204, France; 2 Equipe labellisée Ligue Contre le Cancer, Nice 06204, France; 3 Vall d’Hebron Research Institute (VHIR), Passeig Vall d’Hebron 119-129, Barcelona 08035, Spain; 4 Catalan Institute of Nanoscience and Nanotechnology (ICN2), CSIC and BIST, Campus UAB, Bellaterra, Barcelona 08193, Spain; 5 Institució Catalana de Recerca I Estudis Avançats (ICREA), Barcelona 08010, Spain; 6 Networking Research Centre for Bioengineering, Biomaterials, and Nanomedicine (CIBER-BBN), Instituto de Salud Carlos III, Madrid 28029, Spain; 7 CRCT, Université de Toulouse, Inserm, CNRS, Université Toulouse III-Paul Sabatier, Centre de Recherches en Cancérologie de Toulouse, Toulouse 31100, France; 8 INSERMU955; Unité hémopathies lymphoïdes, Hôpitaux Universitaires Henri Mondor, Assistance publique des Hôpitaux de Paris, Université Paris-Est Créteil; Institut Mondor de Recherche Biomédicale, Créteil F-94010, France; 9 département de pathologie, AP-HP, Groupe hospitalo-universitaire Chenevier Mondor, Créteil F-94010, France; 10 Service Unité Hémopathies Lymphoides, AP-HP, Groupe hospitalo-universitaire Chenevier Mondor, Créteil F-94010, France; 11 CIRI, Université de Lyon; INSERM U1111; ENS de Lyon; University Lyon1; CNRS UMR5308, Lyon 69007, France

**Keywords:** ROS, antioxidant, mitochondria, cerium oxide nanoparticles, T cell lymphoma, cancer, AITL preclinical model

## Abstract

Cancer cell survival and proliferation are correlated with increased metabolic activity and consequent oxidative stress, driving metabolic shifts that interfere with the immune response to malignant cells. This is the case of high-energy-demanding angioimmunoblastic T cell lymphoma (AITL), a highly aggressive cancer with poor survival rates, where malignant CD4+ PD-1^high^ T cells show increased mitochondrial activity and Reactive oxygen species (ROS) accumulation. Here, we report that administration of ROS scavenging cerium oxide (CeO_2_) nanoparticles in an AITL preclinical mouse model leads to their preferential accumulation in the spleen, where the CD4+ PD-1^high^ T cells driving malignancy were significantly reduced. This was accompanied by activation of previously exhausted cytotoxic CD8+ T cells, restoring their potent antitumor function. As a result, survival rates dramatically increase with no observed toxicity to healthy cells or tissues. Overall, it highlights the correlation between increased energy demand, increased mitochondrial mass, increased PD-1 expression, increased ROS production, and immune suppression and how this vicious loop can be stopped by scavenging ROS.

## Graphical abstract

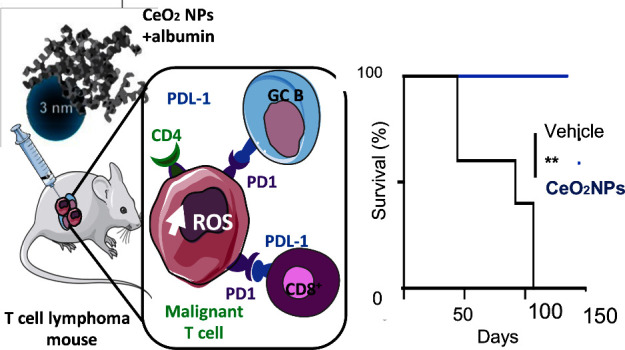

## Introduction

Our body is constantly producing new cells, some of which have the potential to become cancerous. Usually, these cells with damaged DNA repair themselves or die off through apoptosis. If they survive, they are readily eliminated by the immune system, which constantly scans our body for pathogens and malignant cells. Immunosurveillance is the term employed to describe how the host immune system recognizes precancerous cells and destroys them.^1^ Unfortunately, some of these cells become invisible to the immune system by overexpressing membrane cell ligands that act as immune checkpoints, thus preventing immune cells from destroying them. This is the case for a protein known as programmed cell death protein 1 (PD-1) and its ligand (PD-L1), found on the surface of T and B cells, which are key immunosuppressive proteins that inhibit autoimmune responses and maintain central and peripheral immune tolerance, regulating immune homeostasis. Thus, PD-1 works like an ‘identity card’ and prevents cells from being destroyed by cytotoxic CD8+ T cells,^2^ which have their primary role in killing infected and tumoral cells. As abnormally high PD-1 expression on tumor cells mediates tumor immune escape, developing anti-PD-1/PD-L1 antibodies has recently become a hot topic in cancer immunotherapy.^3^

In addition to evading the immune system, cancer cells generate elevated levels of mitochondrial-derived reactive oxygen species (ROS) in the form of highly oxidant free radicals, such as superoxide ions, hydrogen peroxide, and hydroxyls, leading to oxidative stress in the tumor microenvironment and impairing T cell function.^4–7^ Oxidative stress has been postulated to drive permanent activation of immune cells. It leads to their exhaustion and consequent immunosuppression, particularly affecting CD8+ T cells,^8–10^ where they become dysfunctional after reaching the tumor site.^8–10^ This excess mitochondrial ROS production in the tumor microenvironment can be associated with the high energy demand and increased anabolism required for cancer cell rapid growth. Indeed, an increase in energy demand in cancer cells was reported in the 1920s when heightened glucose consumption and lactate production in cancer cells were observed.^11,12^ This phenomenon, later labeled the Warburg effect, describes the metabolic reprogramming of cancer cells that sustains proliferation, accelerates malignant progression, and suppresses immunosurveillance.^1,13–15^ Myriad studies since then have correlated that the most aggressive tumors are characterized by cells with the most elevated ATP production rates.^13^ This relationship between mitochondrial state, oxidative stress, and immune cell function^15–19^ points toward energy metabolism as a relevant therapeutic target.^20^

Given the close correlation between high ROS levels, the metabolic reprogramming of cancer cells, and CD8+ T cell exhaustion and immunosuppression, we hypothesize that ROS scavengers might decrease tumor proliferation rates and malignancy while recovering the immune function to fight cancer. We focus here on ROS scavenger mineral antioxidants, also called nanozymes.^21^ In particular, rare earth cerium oxide (CeO_2_) nanoparticles (NPs), which appear safe and exhibit high ROS scavenging capacity, are known for their biocompatibility, also in their synthesis,^22–24^ with a high degree of versatility.^25^ Initially, CeO_2_ NPs were employed to protect tissues from oxidative stress and radiation-induced damage and were later studied for their immunomodulatory capacities.^26^ This ability stems from defects in their crystalline structure when reaching the nanoscale, creating oxygen vacancies (Ov) at the NP surface, leaving an extra electron behind in a Ce^3+^ ion in the Ce^4+^O^2–^ fcc nanocrystal which can be reoxidized to Ce^4+^ providing an electron to and hydroxyl group transforming it in hydroxide, and reduce again back to Ce^3+^ taking an electron from another hydroxyl group producing molecular oxygen recurrently. This Ce^4+^/Ce^3+^ duality enables the formation and annihilation of Ov at the NP surface, permitting electron exchange with surrounding molecules to scavenge free radicals, catalyzing the degradation of excess ROS without being consumed.^27^

The antioxidant and anti-inflammatory efficacy of CeO_2_ NPs has been tested in a wide variety of preclinical models of diverse pathologies, including cardiac diseases,^28^ brain ischemia,^29^ diabetes,^30^ retinal dysfunction,^31^ liver inflammation,^32^ sepsis,^33^ acute and chronic glaucoma,^34^ and neurodegeneration,^35^ showing therapeutic benefits across all these different cases, demonstrating to be safe to normal tissue and highly soluble in biological media; to present high bioavailability and to disperse well in tissues.^36^ Indeed, the use of CeO_2_ NPs in cancer is not new. For example, pure CeO_2_ NPs have been employed to treat cancers such as hepatocellular carcinoma^37^ and fibrosarcoma.^38^ Also, Sack et al. found that CeO_2_ NPs increased the antitumor activity of doxorubicin in melanoma cells, with synergistic effects on cytotoxicity.^39^ Similarly, CeO_2_ NPs combined with sorafenib were administered to rats bearing hepatocellular carcinomas,^37^ and plant-based synthesized CeO_2_ NPs improved the anticancer effects of Temozolomide in glioblastoma (U87) cells.^40^ Recently, zirconium-doped CeO_2_ NPs have been shown to counteract immunosuppression and enhance immune checkpoint inhibitor efficacy in renal and breast cancer models.^41^ Another innovative application of CeO_2_ NPs comes from Koula et al., who developed CeO_2_ NPs capable of crossing the blood-brain barrier to target gliomas.^42^ This system demonstrated a 3-fold increase in survival in glioma models and reprogrammed tumor-associated macrophages, enhancing T cell activity.

To test the hypothesis of the ROS scavenging effects of CeO_2_ NPs on cancer cells regarding mitochondrial activity, ROS production, immune activation, and survival, we selected a model of a rare peripheral T cell lymphoma called angioimmunoblastic T cell lymphoma (AITL). AITL is recognized as a CD4+ follicular helper T cell disorder known for its anomalous PD-1 expression and high metabolic activity, leading to increased ROS production. AITL is a devastating, highly energy-demanding non-Hodgkin lymphoma, found in the lymphatic system, especially in the spleen, showing increased mitochondrial respiration rates and ROS production together with exhausted CD8+ T cells, incapable of exercising their expected antitumoral activity.^43^ With no adapted treatment, its overall 5 year survival rate is below 30% and fatal relapses are commonly observed.^44^ In this model, aged mice develop the same T cell lymphoma as their human AITL counterparts and represent a valid preclinical disease model,^45^ in which we previously evaluated different novel therapeutic approaches.^45–48^ As in human AITL patients, the model is characterized by CD4+ T cells containing two subsets overexpressing PD-1: the PD-1^low^ subset, which is considered the progenitors of tumor cells, and the PD-1^high^ subset, which is regarded as the driver of AITL malignancy.^46^ Although CD4+ T cells are the drivers of malignancy, they are closely associated with germinal center (GC) B cells, also found in the spleen; their contact is required for mutual survival and growth.

Therefore, the AITL mouse model was preferred for evaluating the ROS scavenging effect on tumor progression and the anticancer immune response.

## Results

First, CeO_2_ NPs were synthesized under sterile conditions using a classical hydrothermal approach. The process involved basic precipitation with sodium citrate serving as both a complexing and stabilizing agent, following a previously established protocol.^49^ In detail, CeCl_3_ was dissolved in a sodium citrate aqueous solution (1:2 molar ratio) before a strong base, such as TMAOH, was added to promote basic precipitation. Thereafter, thermal treatment was applied, followed by purification to remove excess citrate and reaction byproducts, yielding a stable, pale-yellow colloidal solution. NP morphology and size distribution were analyzed by high-angle annular dark field (HAADF) and high-resolution transmission electron microscopy (HRES-TEM), which indicate the formation of nonaggregated quasi-spherical single-crystal 3 ± 1 nm CeO_2_ NPs of high uniformity and narrow size distribution (Figure 1A). HAADF exploited the strong electron scattering on cerium ions due to their high Z number (58). The scattered electrons at high angles come exclusively from Ce, forming element-sensitive images. Additionally, Figure 1B presents the X-ray diffraction (XRD) pattern of the as-synthesized CeO_2_ NPs showing well-defined peaks corresponding to a cubic fluorite (fcc) structure (JCPDS No. 34–0394), confirming the sample as pure single-phase cerium oxide. The broadening of the diffraction peaks indicates a small particle size and/or crystal disorder, produced by defects and impurities, such as Ov and different degrees of structural hydration (*vide infra*). Supposing all the peak broadening is due to finite size effects, the estimated crystalline diameter obtained using the Scherrer equation is approximately 2.7 nm, very close to the one observed by TEM. The 220 peak, intense and well resolved, was chosen for this calculation since the convolution of the 200 and 222 peaks with the 111 and 311 peaks, respectively, distorts peak broadening and, therefore, affects the Full Width at Half-Maximum (FWHM) employed in the equation to estimate the crystalline domain size. Using Bragg’s law, the lattice constant is estimated to be 5.420 Å, slightly higher than the bulk value (5.411 Å).^50^ This can be attributed to a high concentration of Ov, which consequently increases electrostatic repulsion between Ce cations in the crystal when the oxygen between them is removed, leading to a consequent lattice expansion.^51^

**Figure 1:**
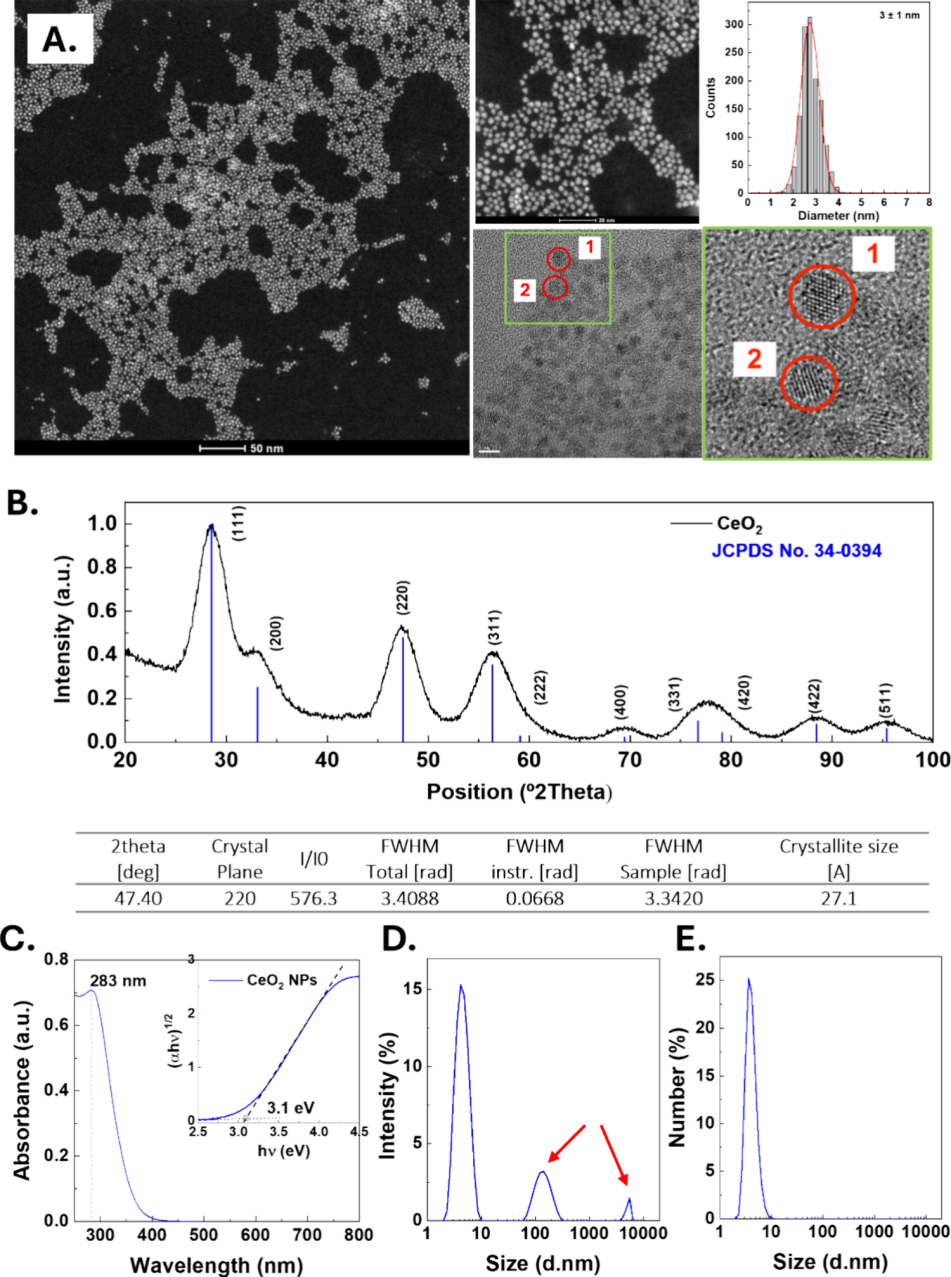
Characterization of CeO_2_ NPs. (A) Representative high annular dark field (HAADF) and high-resolution (HRES) transmission electron microscopy (TEM) images. Scale bars are 50, 20, and 5 nm, respectively. The corresponding size distribution was determined by analyzing more than 1500 particles. The insert provides a zoomed-in view of the crystal planes of CeO_2_ NPs. (B) X-ray diffraction (XRD) pattern of the as-synthesized CeO_2_ NPs and JCPDS 34–0394 standard. (C) UV–vis spectra of CeO_2_ NPs and their representation as Tauc plot from the UV–Vis analysis in the inset were the intersection of the two fitting lines estimates the band gap energy.^55^ (D) DLS spectra of scattered light by intensity and (E) DLS spectra of CeO_2_ NP by number distribution.

Next, we employed UV–vis spectroscopy to determine the presence of CeO_2_ NPs and their band gap. Figure 1C shows the UV–vis extinction spectra, depicting prominent absorption between 250 and 360 nm, with a peak at 283 nm corresponding to CeO_2_ NPs. The absence of light extinction in the visible range indicates the absence of CeO_2_ NP aggregates, which would manifest as broad extinction bands at higher wavelengths (above 400 nm) due to Mie scattering. Tauc plots displaying the UV–Vis extinction spectra relative to the photon energy (Figure 1C, inset) were employed to fit indirect energy transitions. The band gap between the top of the O 2p valence band and the bottom of the conduction band formed by the localized Ce 4f orbitals directly correlates with the concentration of Ov and their concomitant Ce^3+^ ions in the NP. This yielded an optical band gap energy of 3.11 eV (bulk values are reported between 3.15 and 3.20 eV). The band gap of semiconductors is known to expand due to quantum confinement effects when the NP diameter becomes smaller than its Bohr radius, which is 7 nm for CeO_2._ ^52,53^ Since the CeO_2_ NPs in this study meet this condition, an increase in the band gap would typically be expected. This phenomenon is well-documented in other wide band gap semiconductor NPs, such as TiO_2_ or ZnO, where band gap increments of a few eV have been observed.^54^ However, Ov in CeO_2_ leaves behind Ce^3+^ ions with an extra 4f^1^ electron, introducing charge carriers to the conduction band that compensate for the quantum confinement effects, resulting in similar band gaps for bulk and nanometric CeO_2_.

The light scattering contribution due to Rayleigh scattering, when particles are much smaller than the wavelength of the incident radiation, is read by Dynamic Light Scattering (DLS) and translated to a hydrodynamic diameter. Note that the measure is very sensitive to aggregates since the scattering intensity increases with the sixth power of the diameter; therefore, large NPs (or aggregates) would be overrepresented in the distribution and easily detected. In this case, however, the observed peaks at 150 and 6000 nm (red arrows) that appear in the intensity scattering spectra (Figure 1D) are not caused by the presence of a few large NPs but by artifacts resulting from multiple scattering events due to the high concentration of small CeO_2_ NPs needed to obtain a readable signal. Note that if these peaks were made from aggregated CeO_2_ NPs, due to their large density (7.2 g/mL), they would swiftly sediment, and it would be very easy to filter them out of the solution or centrifugate them, which is not the case here, where the apparent aggregates are stable in solution over weeks before and after mild filtration/centrifugation (see SI for a more detailed discussion). Thus, once the scattering intensity distribution is transformed into the frequency of size distribution (number) (Figure 1E), a single representative peak of the NP size distribution is observed and centered at 4 nm, consistent with the “dry” NP radii observed by TEM and X-ray diffraction. Finally, thermogravimetric analysis (TGA) was performed to determine mass losses in the prepared NPs and a signature of the sample composition and NP hydration (Figure S1). This is because at high pH, Ce^3+^ oxidizes and precipitates, first as Ce­(OH)_4_ and then, by condensation (and dehydration), it is transformed into CeO_2_ (Ce­(OH)_4_ → CeO_2_ + 2H_2_O). In industrial processes, the basic precipitation of CeO_2_ is typically followed by a calcination step to complete the Ce^3+^ oxidation and full dehydration of the precipitate, obtaining pure CeO_2_ samples. Under an N_2_ atmosphere, hydrated CeO_2_ starts losing water at around 100–200 °C, and complete dehydration generally occurs between 300–400 °C when structural water is removed and Ce­(OH)_4_ is transformed into CeO_2_. Above 400 °C, there is the formation of fully crystallized CeO_2_. In parallel, it is known that pure sodium citrate dehydrates at 150–250 °C, while at 300–400 °C, citrate anions decompose, releasing CO_2_ and other organic byproducts. Above 500 °C, there is complete decomposition, leading to the formation of sodium carbonate and carbonaceous residues.

Due to the highly nonstoichiometric nature of CeO_2_ at the nanoscale, both Ce^3+^ and Ce^4+^ oxidation states coexist. To evaluate the Ce^3+^/Ce^4+^ ratio in the CeO_2_ NPs, where each Ce^3+^ ion corresponds to an Ov, samples were analyzed using X-ray photoelectron spectroscopy (XPS). A more standard CeO_2_ NP sample with an approximate size of ∼5 nm was prepared in the absence of SC (see ref 37) and was compared with the 3 nm CeO_2_ NP used in this study. XPS analysis provides insights into the first few atomic layers of the material, as the attenuation length of emitted photoelectrons typically ranges from 2 to 5 nm.^56^ Figure 2 displays the XPS survey spectra of the two samples. In the wide-scan spectrum, peaks at approximately 285, 400, and 530 eV correspond to the C 1s, N 1s, and O 1s regions, respectively. Specific Auger transitions are also observed at higher binding energies alongside the characteristic Ce 3d peaks around 900 eV. The Ce 3d core-level spectra were fitted and transformed into deconvoluted Gaussian–Lorentzian peaks, revealing six peaks corresponding to Ce^4+^, which represent three pairs of spin–orbit doublets (V, V″, V‴, U, U″, and U‴), and four peaks (two doublets) corresponding to Ce^3+^ (V0, V′, U0, and U′). These assignments are based on peak positions reported by Mullins et al.,^57^ where U and V denote the 3d_3_/_2_ and 3d_5_/_2_ spin–orbit components, respectively. By quantifying the contributions from each oxidation state in the measured conditions, the concentration of Ce^3+^ ions in our 3 nm sample is 42.1%. This exceptionally high Ce^3+^ concentration, reflecting a high density of oxygen vacancies, is among the highest reported for pure CeO_2_.^58^ The result is consistent with the small size of the NPs, as reduced particle dimensions lead to a higher surface-to-volume ratio and increased lattice strain, both of which favor the formation of oxygen vacancies. In the 5 nm control CeO_2_ NPs, the Ce^3+^ concentration is lower, about 23.2%, which agrees with its bigger size. Notably, for the 3 nm CeO_2_ NPs, the O 1s region exhibits significantly stronger peaks due to the presence of citrate. Na 1s peak appears at 1071 eV. The C peaks are also attributed to the presence of citrate. In addition, in the O 1s spectrum, the CeO_2_ NPs exhibit three components fitted with Gaussian peaks and labeled O1, O2, and O3. The peak at lower photoelectron binding energy (O1) is attributed to lattice oxygen species, in our case, at 529.7 eV. In contrast, those at higher photoelectron binding energies (O2 and O3) are usually attributed to the weakly absorbed oxygen on the CeO_2_ surface.

**Figure 2:**
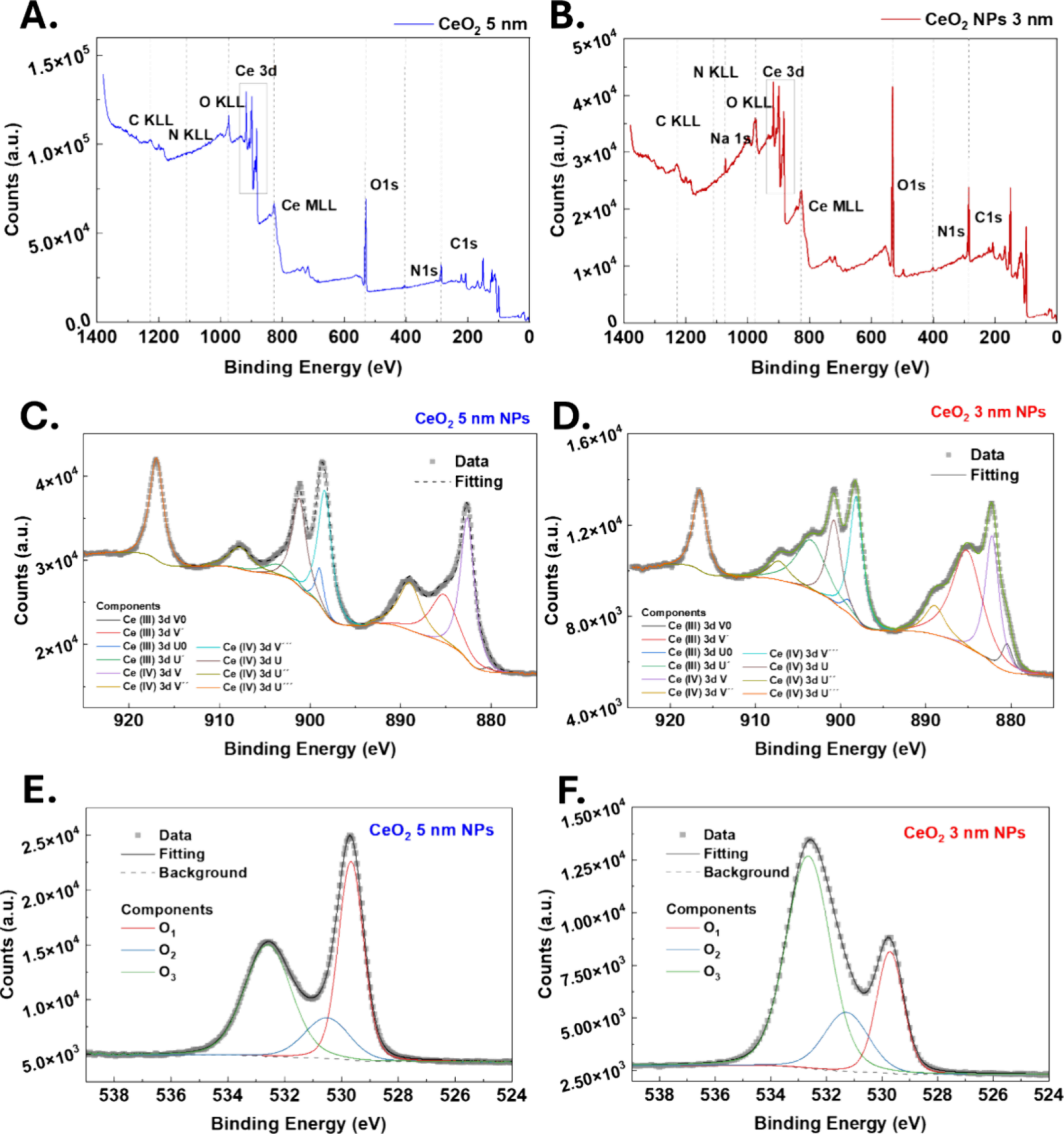
Survey XPS spectra of 5 nm CeO_2_–NPs (A) and CeO_2_–SC 3 nm NPs (B). Both spectra exhibit comparable features. In the wide-scan spectrum, peaks at approximately 285, 400, and 530 eV correspond to the C 1s, N 1s, and O 1s regions, respectively. Specific Auger transitions are also observed at higher binding energies alongside the characteristic Ce 3d peaks. Ce 3d XPS spectra of CeO_2_ 5 nm NPs (C) and CeO_2_ 3 nm NPs (D). Peaks corresponding to Ce^3+^ (V0, V′, U0, and U′) and Ce^4+^ (V, V″, V‴, U, U″, and U‴) components are labeled, where V and U indicate the 3d_5_/_2_ and 3d_3_/_2_ spin–orbit levels, respectively. The experimental spectrum is shown as a gray dotted line, and the fitted spectrum is a black-solid line. O 1s XPS survey spectra of CeO_2_ 5 nm NPs (E) and CeO_2_ 3 nm NPs (F). Finally, the peak at higher binding energies (O_3_), typically observed between 532.5 and 533.0 eV, corresponds to water molecules irreversibly adsorbed on the NPs.

Before the application of NPs to biological media, the interaction of NPs with proteins is fundamental. High-energy NP surfaces readily interact with proteins when they enter physiological media, forming a coating called the protein corona (PC), which not only prevents NP aggregation and ensures biocompatibility but also determines their biological identity.^59^ During the process, NPs and proteins spontaneously interact, first electrostatically, and a transient soft PC forms, which then evolves due to molecular rearrangements and protein crowding effects toward a more permanent, hard PC.^59^

Thus, to avoid uncontrolled PC formation when CeO_2_ NPs are administered, NPs were conjugated with murine serum albumin (MSA) to form an albumin-PC.^60^ Purified CeO_2_ NPs were incubated with an excess of MSA (1:10) for 24 h at 4 °C in 10 mM phosphate buffer (PB). Note that citrate is not strongly bonded to the NP, and albumin easily displaces it at the NP surface.^60^ It is important to note that an incomplete PC is formed when NPs are smaller than proteins. In this case, stable single NP-protein conjugates are expected to form with 3 nm CeO2 NPs and 8 nm MSA. On one hand, conjugation is performed with excess albumin to prevent multiple NPs from binding to a single protein. On the other hand, the presence of a protein at the NP surface hinders the absorption of another one, resulting in single CeO_2_ NP-MSA conjugates.^61^ As a result, the prepared samples contained approximately 10% albumin conjugated to the CeO_2_ NPs, with the remainder of the albumin remaining unconjugated.

The formed CeO_2_NP-MSA solution displays UV–vis absorption spectra similar to those of the nonconjugated CeO_2_ NPs (Figure 3A), and the CeO_2_ NPs become optically indiscernible from the albumin, even after filtration, suggesting their conjugation (Figure 3E, F). The spectrum of MSA alone, at the same concentration used in the working samples (1 mg/mL, pH 7.5), is also displayed. The energy band gap value of the CeO_2_ NPs after conjugation with MSA is identical to that obtained before conjugation (Figure 3A, inset), suggesting that the presence of MSA does not alter the electronic structure of the NPs. The DLS diameter of the mixture is similar to that of MSA in solution at the working concentrations, and the individual CeO_2_ NPs cannot be observed (Figure 3B). The absence of any other peak when conjugating the NPs indicates both the lack of CeO_2_ or protein aggregates and the absence of individual CeO_2_ NPs, suggesting its absorption to albumin without significantly changing its hydrodynamic diameter, forming single CeO_2_ NP–Protein conjugates; otherwise, unconjugated NPs in DMEM result in large aggregates (Figure 3C). The Z potential confirmed the formation of the CeO_2_ NP-MSA conjugates (Figure 3D), showing surface charge values for the conjugates in 10 mM PB (conductivity 0.27 mV) of −11 mV, which is a mix of the Z potential of the individual components (CeO_2_ NPs −40 mV, and MSA −5 mV), indicating the formation of objects partially coated by proteins as depicted in Figure 3D (otherwise, if MSA fully coated the NP, the Z potential would be that of the MSA).^61^ Furthermore, we performed filtration–centrifugation experiments before and after albuminization (Figure 3E, F) to discern CeO_2_ NPs from MSA in our samples. Samples were centrifuged 3 times for 10 min at 2.500g with centricons of 50 kDa (pore size 6 nm), which allows the NPs to cross the filter but not the albumin. UV–visible spectra confirmed how MSA prevents the CeO_2_ NPs from being filtered, corroborating its conjugation (Figure 3E).

**Figure 3:**
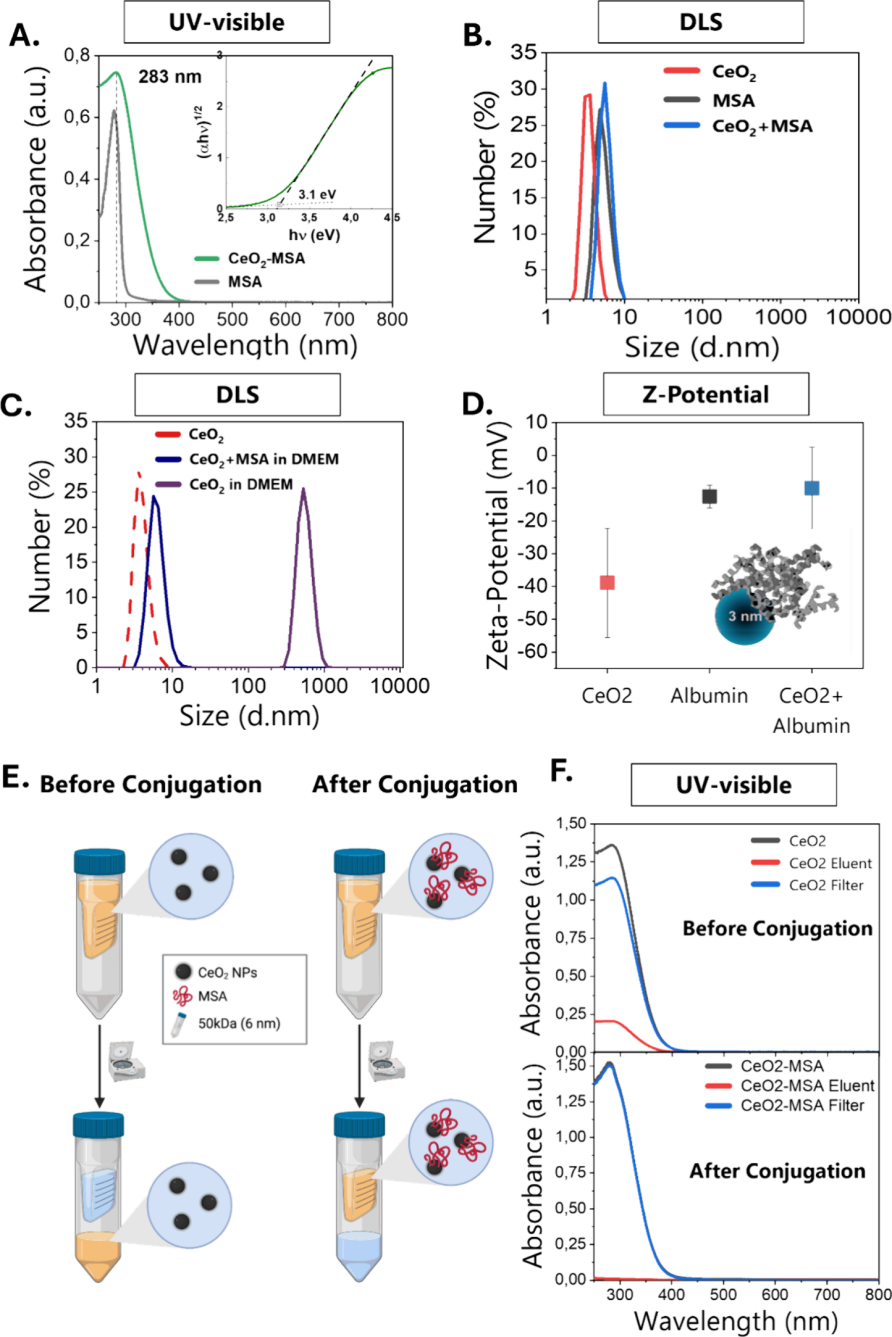
Characterization of CeO_2_-MSA conjugates. (A) UV–vis spectra of CeO_2_-MSA conjugates (green) and free MSA (gray), with the inset showing the Tauc plot, indicating no significant changes in the electronic structure of NPs after conjugation. (B) Hydrodynamic size distribution was measured by dynamic light scattering (DLS) before and after conjugation, revealing an increased NP diameter due to protein binding. (C) Stability assessment of CeO_2_ NPs and CeO_2_-MSA NPs dispersed in cell culture medium (DMEM + 10% FBS) for 24 h. Bare CeO_2_ NPs aggregate under physiological conditions, while MSA conjugation prevents aggregation. (D) Zeta potential measurements before and after conjugation reveal a shift in surface charge to −11 mV, reflecting a mix of the Z potential of CeO_2_ (−40 mV) and MSA (−5 mV), confirming the partial protein coating. (E, F) Protein corona (PC) characterization using centrifugal filtration with 50 kDa (6 nm pore size) filter. The filter retained CeO_2_-MSA NPs, while unconjugated CeO_2_ NPs passed through, as confirmed by UV–vis analysis.

As shown in the Tauc plots, XPS data also indicate that the conjugation with MSA does not change the electronic structure of the CeO_2_ NPs (Figure S2). After albuminization, the cerium XPS peaks of the 5 and 3 nm NPs remained unaltered, showing similar Ce^3+^/Ce^4+^ ratios. At the same time, the C 1s and N 1s regions displayed significantly stronger peaks, confirming the presence of MSA at the NP surface. The presence of more or less of Ov at the NP surface did not appreciably affect protein absorption in the 5 and 3 nm CeO_2_ NPs (Figure S2). Furthermore, we also observed the dispersion of the NPs onto the microscopy substrate before and after conjugation with MSA, which reveals aspects of the surface chemistry of NPs.^62^ As shown in Figure 4, the distribution of conjugated NPs onto the substrate at similar densities shows very different patterns. The CeO_2_-MSA conjugates (Figure 4A) are more separated, with less connectivity and much higher lacunarity than the citrate-stabilized CeO_2_ NPs (Figure 4B), which shows a higher degree of aggregation. This depends on NP-substrate affinity; albumin has a higher affinity for the carbon film where the samples are dispersed for TEM observation, and albumin also protects the material from NP aggregation. In the case of conjugated NPs, discrete aggregates are observed, suggesting the partial coating of the NP surface and confirming previous results.

**Figure 4:**
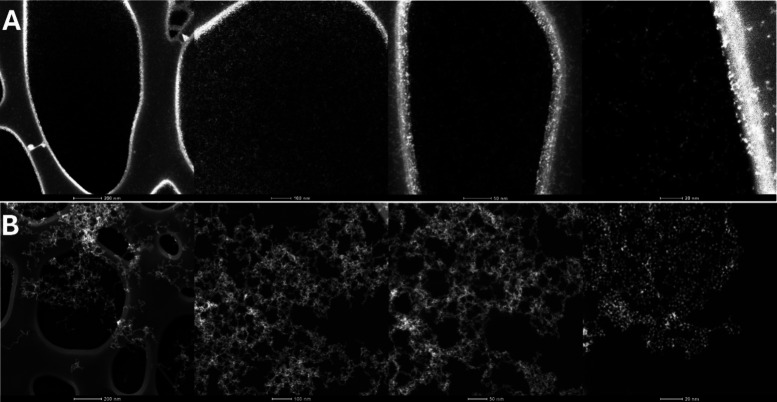
HAADF-TEM images of conjugated CeO_2_ NPs-MSA (A) and unconjugated CeO_2_ NPs (B) samples after drop casting 10 μL of the solution onto ultrathin carbon films supported onto holey carbon films onto a Cu grid. Scale bars are left to right, 200 nm, 100 nm, 50 and 20 nm, showing different textures depending on their different surface states onto the same substrates.

As a final consideration, the presence of the albumin at the NP surface has been reported to have modest effects on decreasing the catalytic activity of the CeO_2_ NPs,^63^ which is compensated by improving biocompatibility, avoiding NP aggregation in physiological media, and preventing renal filtration (as it is common for objects smaller than 6 nm).^64^ Thus, the ROS scavenging capacity of the 3 nm CeO_2_ NPs before and after albuminization was determined using the Amplex red hydrogen peroxide assay kit. Fluorescence was measured with excitation/emission wavelengths set at 540/590 nm, and a modest decrease in catalytic activity was observed (Figure 5).

**Figure 5:**
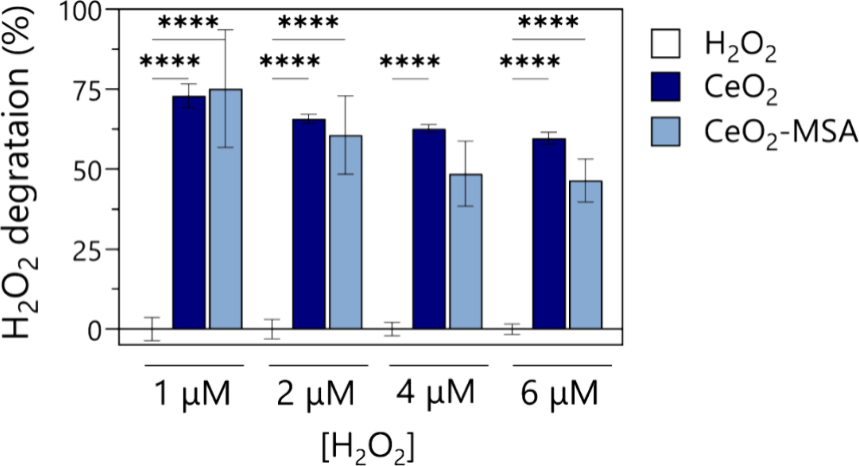
Catalytic activity of CeO_2_NPs evaluated at a concentration of 50 μg/mL following a 40 min incubation with varying H_2_O_2_ concentrations (1, 2, 4, and 6 μM). Scavenging capacity was assessed depending on the surface coating (bare or MSA-conjugated). An Amplex red hydrogen peroxide kit was employed to measure H_2_O_2_ levels according to the manufacturer’s instructions. Data presented as mean ± SEM (*n* = 3; *p < 0.05, **p < 0.01, ***p < 0.001, ****p < 0.0001).

Once the NPs were prepared, purified, albuminized, and thoroughly characterized, we studied their biological impact. First, we assessed the metabolic state of the cancer cells in the AITL mouse model of the study. In AITL disease, malignant T cells show a significant upregulation of the expression of 52 genes implicated in mitochondrial respiration as well as the expression of 70 genes related to the electron transport chain (ETC) gene signature,^46^ reflecting their metabolic reprogramming (Figure S2). FACS analysis of mice AITL CD4+ PD-1^low^, CD4+ PD-1^high^ cells, AITL CD8+ T cells, and WT CD4+ and CD8+ T cells was performed by staining for mitochondrial content using Mitotracker green (MTG) and ROS levels using the CellROX probe (Figure 6A). The results directly correlated with increased mitochondrial mass, ROS production, and PD-1 overexpression. In contrast, CD8+ T cells showed no significant increase in mitochondrial mass but exhibited elevated ROS production, a feature of exhausted CD8+ T cells associated with impaired functionality (Figure 6B).^65^ After staining with MTG, confocal microscopy confirmed that the mitochondrial mass increase could be mainly attributed to the malignant subset of CD4+ PD-1^high^ cells (Figure 6C). To correlate and validate this high level of mitochondrial mass and ROS production in human patients, we generated Affymetrix data for CD4+ PD-1^high^ T cells isolated from biopsies of 6 different AITL patients. The resulting Gene Set Enrichment Analysis (GSEA) heatmap confirmed a statistically significant gene signature enrichment associated with increased mitochondrial and ROS production in neoplastic CD4+ T cells. Notably, this signature was not enriched in noncancerogenic CD4+ T cell subtypes, such as central and effector memory, naïve, or stem cell memory cells of the same patients (Figure S3).

**Figure 6:**
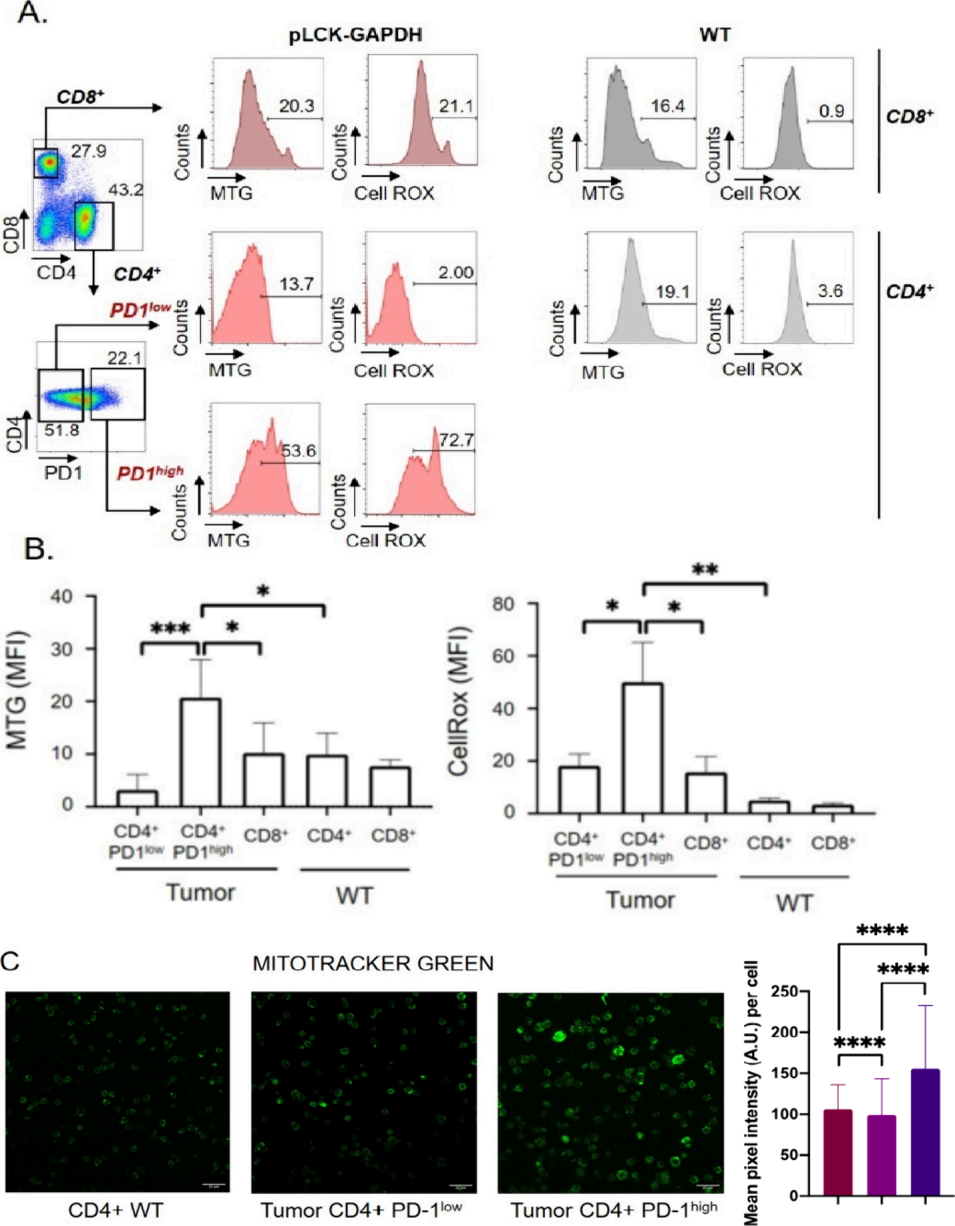
Murine AITL malignant CD4 T cells exhibit elevated mitochondrial mass and ROS levels as compared to WT CD4+ T cells. (A, B) FACS analysis was performed on AITL CD8+, CD4+PD-1^high^ cells and CD4+PD-1^low^ T cells, as well as WT CD4+ and CD8+ T cells, stained for mitochondrial content using Mitotracker Green (MTG) and ROS levels using a CellROX probe. Data are summarized in panels (A) and (B). Panel (A) shows the histograms, and panel (B) shows the mean ± SD (tumor *n* = 5, WT *n* = 4, ***p < 0.001,). (C) Representative confocal microscope images of live WT CD4+ splenocytes and isolated Plck-GAPDH CD4+PD-1^high^ cells and CD4+PD-1^low^ cells stained for mitochondria by Mitotracker Green. Quantification of the signal is shown on the left. (order: CD4+ WT, CD4+PD-1^low^and CD4+PD-1^high^ AITL cells)

Next, we administered CeO_2_-MSA NPs to healthy mice and searched for potential toxicity effects. Nonaggregated CeO_2_ NPs are considered relatively safe and do not affect basal ROS homeostasis. Seal et al.^66^ have already evaluated their safety and inability to scavenge basal physiological ROS, which is essential for DNA transcription and other molecular processes required for normal cell functioning. Since then, the proper use of CeO_2_ NPs in biomedical applications has consistently shown their innocuity in both in vitro and in vivo models,^36^ except at excessively high doses, where large aggregates may form, or when NPs are contaminated.^67^ When preparing the treatment to be applied, we observed that in the scientific literature, therapeutic effects are observed with doses ranging from 50 to 250 μg of cerium per gram of tissue.^27^ Accordingly, we planned to inoculate 400 μg per mouse (mice weigh around 25 g, with liver weights of 1–2 g and spleen weighing 0.1–0.2 g, corresponding to a dose of 16 mg/kg body weight). Before administration, the colloidal solution was rigorously tested for endotoxins.^68^ Considering that CeO_2_ NPs do not get metabolized during their therapeutic action and that they tend to accumulate in the liver and spleen, we scheduled the therapeutic dose to be administered biweekly for two months (5 doses of 80 μL, each containing 1 mg/mL CeO_2_ NPs conjugated with 10 mg/mL MSA in 10 mM phosphate buffer). This regimen aimed to enhance CeO_2_ NPs embedding in the spleen, promote a more uniform distribution, and minimize the risk of infusion reactions. Infusion reactions are complex, immune-mediated side effects occurring within minutes to hours following the intravenous administration of therapeutic doses. While not exclusive to nanomedicines, these reactions are particularly relevant for NPs, which, due to their size similar to that of proteins and viruses, are especially targeted by the immune system.^69^

First, to evaluate the safety and toxicity of the NPs, healthy NSG mice were treated IV with either the CeO_2_ NP-MSA solution or the vehicle (10 mM PB supplemented with 10 mg/mL MSA). During the duration of the experiment, the weight and behavior of mice were monitored weekly. The spleen, kidneys, lungs, liver, stomach, and intestines were examined at sacrifice, and no signs of inflammation or abnormalities were observed (Figure S4). In addition, there were no signs of abnormal respiration, trembling, curving of the back, weight loss, or changes in mood or behavior. At the same time, animals maintained normal reactivity to stimuli and exhibited a typical mobility in the cage.

Next, we treated the AITL mice and conducted the experiment for 135 days (Figure 7A). The biodistribution of cerium was determined on the day of sacrifice (Figure 7B). The concentration of cerium 65 days after the last CeO_2_ NP injection was still significant in the liver and spleen, with detectable levels in the lungs and trace amounts in the kidneys. Moreover, cerium was not detected in the blood or brain (data not shown). Recently, the accumulation and excretion of CeO_2_ NPs similar to those used in this study were reported following a single injection in healthy mice at a dose of 7.2 mg/kg body weight. In that case, CeO_2_ NPs accumulated majorly in the liver (with over 80% of the injected dose) and spleen (with the remaining injected dose) in a few hours after injection, exhibiting long-term organ retention and slow excretion.^49^ The accumulation of CeO_2_ NPs in the liver and spleen has been previously reported, even in the case of disease models such as hepatocellular carcinoma.^36^ This is due to the structure of blood vessels in these organs, which are called fenestrated blood vessels, with large pores to increase the flow of nutrients, waste and other substances, that favor the accumulation of NPs in these organs, as previously described for small CeO_2_ NPs.^49,70^ From there, cerium is gradually excreted through the hepatobiliary route as NPs, and through the urinary tract after NP disintegration as ions, with 60% of the injected mass eliminated 100 days after injection in healthy mice.^49^ It is worth noting that despite a general belief that NPs are as persistent as their bulk counterparts, smaller NPs tend to be relatively unstable and short-lived due to their high surface energy.^71^ In the case of CeO_2_, at neutral pH and in reducing in vivo conditions, the thermodynamic fate of Ce^4+^ is to become water-soluble Ce^3+^, as the Pourbaix diagram shows.^72^ Thus, small CeO_2_ NPs slowly dissolve in vivo and transform into relatively safe Ce^3+^ ions excreted through the urine after a short residence in the lungs.^49^ This dissolution process is slow, with a reported duration of several months.^49,70,73^ In contrast, bulk ceria remains highly stable due to the substantial activation energy required for CeO_2_ dissolution;^74^ this energy barrier is significantly reduced when the NP size is minimized to just a few nm. In addition, these ultrasmall NPs already display a high ratio of Ce^3+^ ions in the CeO_2_ crystal, favoring its dissolution.^75^ The biodistribution pattern at 135 days is similar to previous reports in healthy models.^49^ In addition, while in the former study, up to 60% of the injected dose was excreted in 100 days, in our current study, a little more than 10% of the injected dose was retained 65 days after the last CeO_2_ NP injection, suggesting faster processing of the NPs in the tumoral spleen.

**Figure 7:**
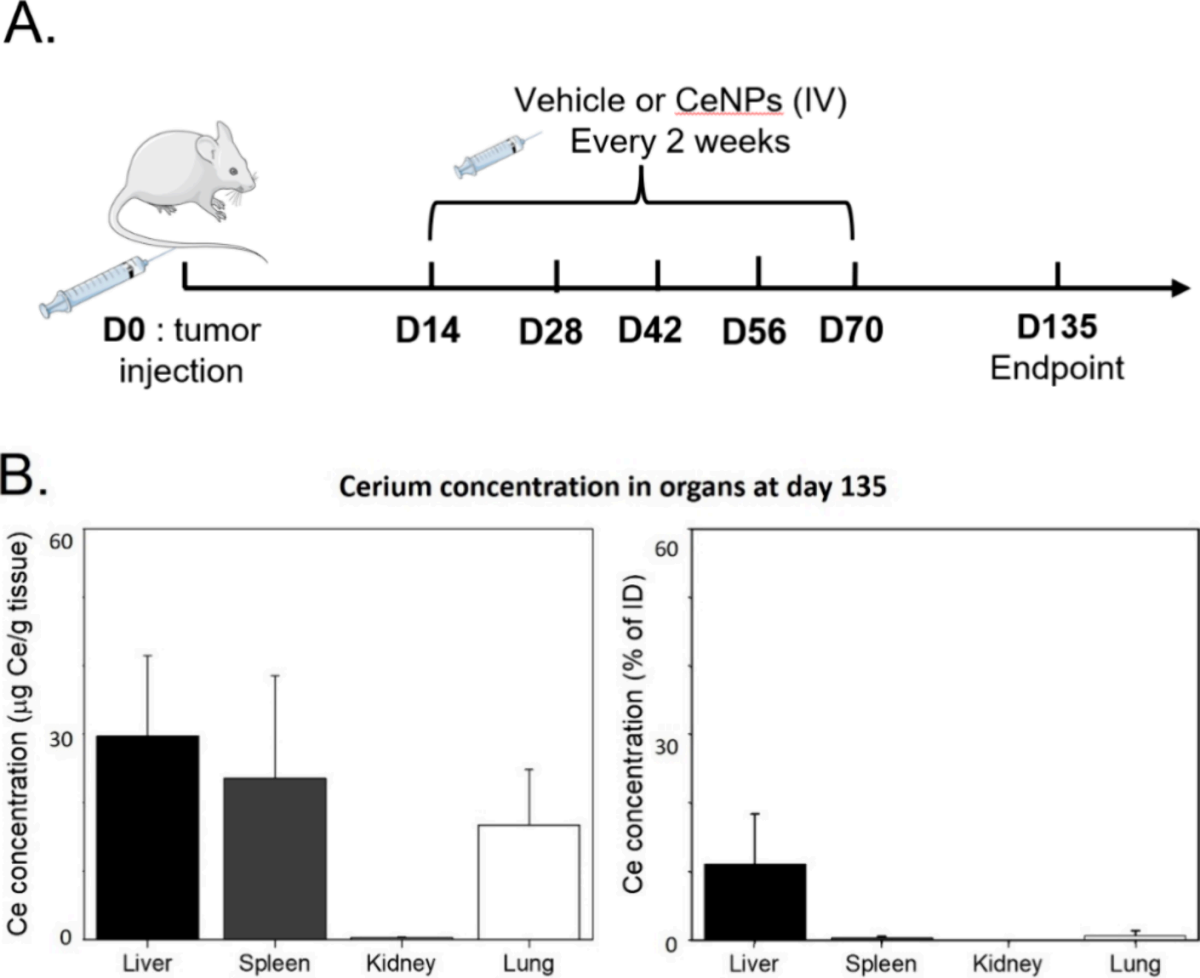
Biodistribution of Cerium 65 days after the last CeO_2_NPs IV administration in a preclinical AITL mouse model. (A) Splenic lymphoma cells were intravenously injected into recipient mice (*n* = 11). Fourteen days postinjection, mice were treated with ROS scavenger CeO_2_ NPs via IV administration of an 80 μg CeO_2_NPs-MSA solution (*n* = 6) or vehicle (10 mM PB supplemented with 10 mg/mL MSA) (*n* = 5). Mice were sacrificed at the experimental end point or 135 days post-transplant. (B) Elemental analysis by inductively coupled plasma mass spectrometry (ICP-MS) of cerium retention in mouse tissues 65 days after the last CeO_2_ NP injection.

Once the presence of cerium was confirmed, spleens were extracted at sacrifice and splenocytes were isolated for analysis. Flow cytometry of CD4+ and CD8+ T cell populations in CeO_2_NP-treated AITL mice revealed a correlation between reduced ROS levels and decreased mitochondrial content (Figure 8A). These beneficial effects of CeO_2_ NPs treatment were also associated with a significant reduction in CD4+PD-1^high^ malignant T cells and associated germinal center (GC) B cells (Figure 8B). The reduction of excess ROS also correlated with reinstalling CD8+ cytotoxicity, as evidenced by the increased production of interferon-γ (INFγ), granzyme B, and perforin (Figure 8C). IFN-γ plays a crucial role in immune activation against cancer cells, serving as a key effector molecule in immunosurveillance.^76^ Indeed, IFN-γ has been shown to protect the host against the growth of transplanted tumors.^77–79^ Analogously, studies have demonstrated that fibrosarcoma grows faster and more efficiently in mice treated with IFN-γ-specific monoclonal antibodies.^77^ In addition, granzyme B, a marker of activated cytotoxic T cells, is secreted alongside perforin, a pore-forming protein, to mediate apoptosis in target cells, further supporting the restoration of antitumoral immune function in treated mice. Most importantly, CeO_2_ NPs treatment significantly increased the survival of AITL tumor-bearing mice (Figure 8D). A reduced mitochondrial mass, decreased ROS production, recovery of the anticancer immune response, and elimination of CD4+ PD-1^high^ T cells accompanied this. Indeed, all AITL mice treated with the vehicle succumbed to lymphoma progression by day 105, whereas all the CeO_2_ NP-treated AITL mice survived an additional month, during which no signs of tumor recurrence were observed. (Figure 8D).

**Figure 8:**
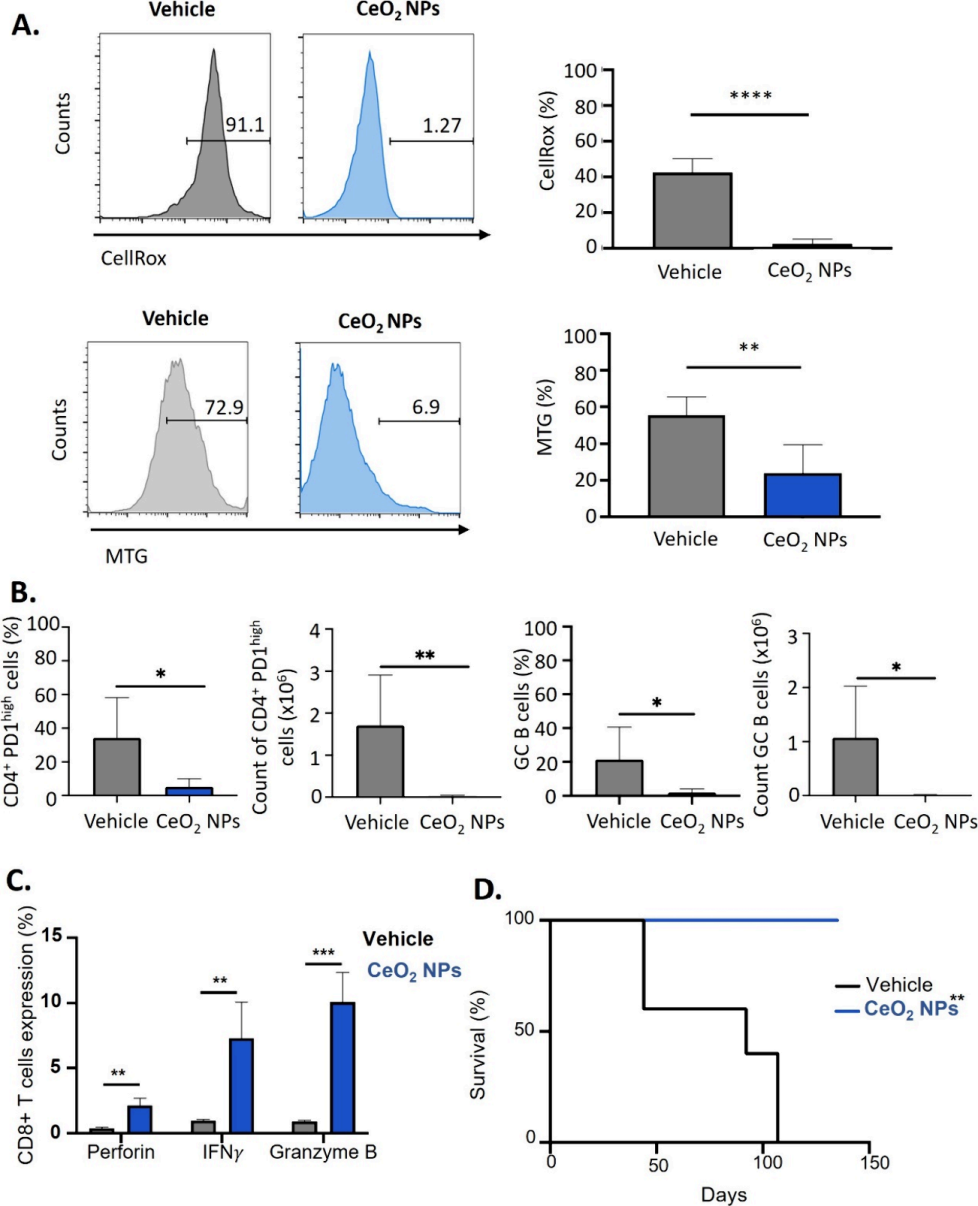
Effect of CeO_2_NPs treatment on Mitochondria, ROS, and malignant T cell survival in the preclinical AITL mouse model. Mice were treated and sacrificed as in Figure 3A. (A) FACS analysis of CD4+ T cells stained for ROS content by the CellROX probe in the spleen of the indicated treatment groups at sacrifice. Data are summarized in the histogram (left) and shown as mean ± SD (right) (vehicle, *n* = 4; CeO_2_ NPs, *n* = 6; ****p < 0.0001). (B) FACS analysis of CD4+ T cells stained for mitochondrial content with MitoTracker green (MTG) in the spleen of the indicated treatment groups at sacrifice. Data are summarized in the histogram (left) and shown as mean ± SD (right); (vehicle, *n* = 5; CeO_2_ NPs, *n* = 6; **p < 0.01). (C) FACS analysis of the percentage and count of CD4+ PD-1^high^ cells on total CD4+ T cells in the spleen of the indicated treatment groups at sacrifice (left). FACS analysis of the percentage and counts of GC B cells (GL-7+ CD95+) on total B cells in the spleen of the indicated treatment groups at sacrifice (right). Data are shown as mean ± SD (vehicle, *n* = 5; CeO_2_ NPs, *n* = 6; *p < 0.05). (D) Splenocytes isolated from CeO_2_ NPs or vehicle-treated AITL mice were activated for 6 h with PMA/ionomycin in the presence of Golgi-stop, followed by surface staining for CD8+ and intracellular staining for INFγ, perforin, and granzyme B. Data are shown as mean ± SD (vehicle, *n* = 4; CeO_2_ NPs, *n* = 3; **p < 0.01, ***p < 0.001, multiple *t* test).

An additional experiment was performed to validate these observations. Now, the treatment was performed ex vivo in lymphoma biopsies obtained from AITL mice to assess the effect of CeO_2_ NPs on cancerous splenocytes directly. Animals were sacrificed 2 weeks after AITL development, and splenocytes were isolated. Cells were treated with CeO_2_ NPs at 50 or 100 μg/mL (in 10 mM PB with 10 mg/mL MSA) or with vehicle (10 mM PB supplemented with 10 mg/mL MSA). Day 3 postincubation, FACS analysis of % of CD4+ PD-1^high^ cells and the % of CellROX+ CD4 T cells showed a significant decrease in the number of CD4+ PD-1^high^ malignant T cells (Figure 9A) due to ROS scavenging (Figure 9B) as compared to vehicle, confirming in vivo AITL mice results.

**Figure 9:**
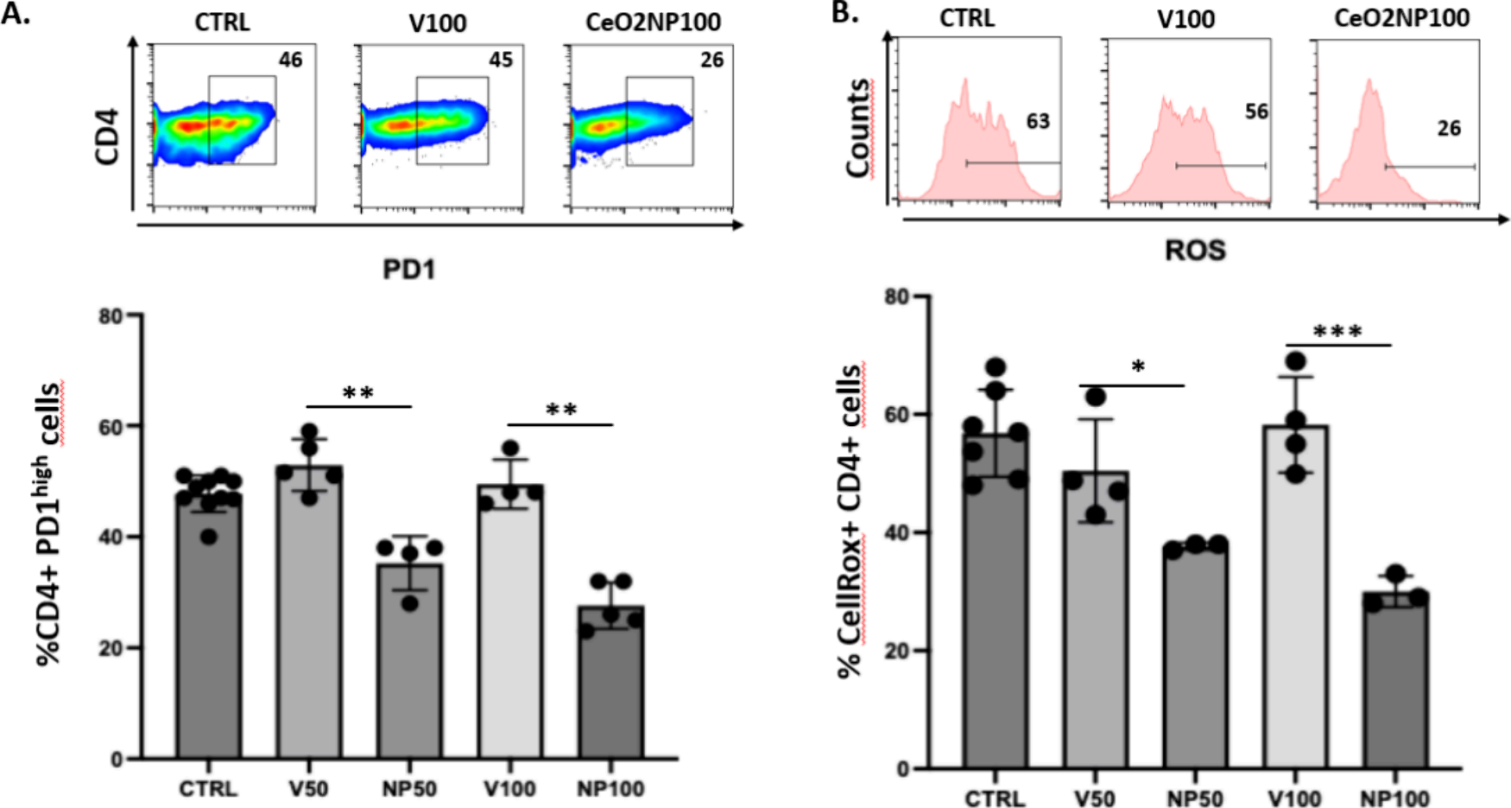
Treatment of T cell lymphoma biopsies with CeO_2_NPs results in the elimination of CD4+ PD-1^high^ malignant T cells due to ROS scavenging. Splenocytes from AITL mice were incubated with medium (CTRL) alone, supplemented with the vehicle (10 mM PB supplemented with 10 mg/mL MSA) or CeO_2_ NPs at 50 or 100 μg/mL (10 mM PB supplemented with 10 mg/mL MSA). Day 3 postincubation, FACS analysis of % of CD4+ PD-1^high^ cells (A) and the % of CellROX+ CD4+ cells (B) per total CD4+ T cells. (mean ± SD; For (A) CTRL, *n* = 8; V50, *n* = 5, NP50, *n* = 4; V100, *n* = 4; NP100, *n* = 4) and for (B) CTRL, *n* = 7; V50, *n* = 4, NP50, *n* = 3; V100, *n* = 4; NP100, *n* = 3). For (A,B), the following gating strategy was applied: gating on single cells was followed by gating on living cells. Then, in a plot of CD4 versus CD8 T cells, the CD4+ cells were gated and in (A) plotted against PD-1 staining or in (B) plotted in a histogram against ROS levels.

## Discussion

Growing evidence highlights the critical role of cancer metabolism in supporting tumor cell survival and proliferation while also influencing antitumor immune responses.^80^ The increased ROS production shows that cancer cells cope with an anomalous increased anti-ROS defense, which promotes chronic oxidative stress in the tumor microenvironment, ultimately leading to immune cell exhaustion and loss of immunosurveillance capacities. Additionally, the overexpression of immune checkpoint molecules, such as PD-1, further facilitates the tumor’s escape from immune detection, promoting its proliferation and migration.

In a previous study, AITL cells showing increased metabolism allowed us to evaluate the effect of mitochondrial electron chain inhibitors, such as Complex I inhibitors metformin and IACS.^46^ Metformin, a widely used drug to treat diabetes, not only improved the survival in the AITL mouse model but also induced the reactivation of cytotoxic CD8+ T cells. This was expected, as Complex I inhibition reduces mitochondrial activity and consequently diminishes mitochondrial ROS production in the AITL CD4+ T cells.^46^ These results highlight the critical roles of metabolic reprogramming and immune modulation in improving cancer therapy. As opposed to the direct cytotoxic effects observed in traditional chemotherapy and radiotherapy, alterations in cancer cell metabolism can significantly reduce tumor malignancy and restore immune function, facilitating tumor elimination. In particular, the remodelling of the metabolic environment in the spleen with CeO_2_ NPs reactivated the tumor-suppressed immune cell activity, initiating an immunotherapeutic effect through antioxidant mechanisms. Thus, mineral antioxidants like CeO_2_ NPs are well-suited for combination with other immunotherapies. However, while CeO_2_ NPs may refrain from tumoral cell aggressivity, their ROS-scavenging^81^ properties can protect cancer cells from apoptosis induced by radio- and chemotherapy. Therefore, CeO_2_ NPs should be explored not for concurrent use with traditional radio- and chemotherapy but rather as a post-treatment strategy to restore immunosurveillance following ROS-induced damage.

Recently, we confirmed that anti-PD-1 immunotherapy significantly prolonged survival in AITL mice by selectively eliminating CD4+PD-1^high^ malignant cells and restoring CD8+ cytotoxicity.^48^ Interestingly, engaging PD-1 induces signaling that limits glucose uptake and glycolytic flux in T cells, directly affecting their metabolism. Our findings also revealed increased lipid metabolism in the CD4+PD-1^high^ malignant cells within the AILT tumor, contributing to the TCA cycle and mitochondrial respiration.^29^ Furthermore, we also confirmed that elevated PD-1 levels correlated with mitochondrial activation are also observed in AITL patients.^46^ In this sense, in vivo chimeric antigen receptor (CAR) therapy^45^ demonstrated disease control by selective removal of the malignant anti-CD4 T cells and reactivation of the CD8+ cytotoxic T cells by the CAR T cells. Therefore, combining CAR T and CeO_2_ NPs therapy might improve the disease outcomes even more. Finally, removal of the GC B cells, which accompany the malignant CD4+ T PD-1^high^ cells in the tumor, might be achieved using anti-CD20 immunotherapy, as CD20 is highly expressed on GC B cells. Since the malignant CD4+ T cells depend on GC B cells for growth and survival, eliminating these B cells via anti-CD20 immunotherapy might reduce tumor development, while CeO_2_ NPs help preserve immunosurveillance. In other words, combining antioxidants with immunotherapy has the potential to provide significant benefits for AITL patients. This is of high importance because single-treatment therapy has proven not to be sufficient to combat this aggressive T cell lymphoma.

## Conclusions

The administered anti-ROS CeO_2_ NPs passively accumulated in the liver and spleen and were still present 65 days after the last administration (10% of the injected dose). Significantly different from traditional small molecules, NP pharmacokinetics strongly depends on the size, shape, surface functionalization, and aggregation state of NPs. Their colloidal properties influence their biodistribution, accumulation, transformations, and excretion profile, hence, their efficacy and safety. This study demonstrates that the small size of these particles (3 nm) and their high stability in physiological media (no aggregation) are safe and do not induce any noticeable effect in healthy cultures and animal models while significantly enhancing their in vivo antioxidant capacity, thanks to their higher surface-to-volume ratio and increased number of oxygen vacancies. Moreover, their small size facilitated their slow dissolution, promoting their clearance through the hepatobiliary and urinary routes,^49^ leaving the body with restored immunosurveillance. Beyond the expected decrease in proliferation rates, interfering with excess ROS resulted in tumor elimination. Interestingly, while the mitochondrial mass remains stable, we observe a significant reduction in the CD8+ T cell ROS levels. Together with that, we observe a remarkable increase in cytokine production activity (perforin, IFNγ, and granzyme B) accompanied by the progressive decrease of PD-1^high^ cells and an excess of GC B cells.

## Methods

### Synthesis and Characterization of CeO_2_ NPs

Three nm CeO_2_ NPs were synthesized using a wet chemistry method based on the basic precipitation of cerium­(III) chloride heptahydrate (CeCl_3_•7H_2_O; Sigma-Aldrich, #228931) in the presence of sodium citrate (SC; sodium citrate tribasic dihydrate; Sigma-Aldrich, #S4641). Specifically, a 50 mL solution of tetramethylammonium hydroxide (TMAOH; Acros-organics, A0353800) (81 mM) was added to a 100 mL solution containing CeCl_3_ (15 mM) and SC (30 mM). This resulted in final concentrations of 27 mM TMAOH, 10 mM CeCl_3_, and 20 mM SC. The reaction mixture was stirred overnight at room temperature. Subsequently, the mixture was transferred to a 250 mL three-necked round-bottom flask and subjected to reflux at 100 °C for 4 h. This process yielded a stable, well-dispersed solution of 3 nm nanoceria at a concentration of 1.72 mg/mL CeO_2_. Prior to use, the nanoparticle solution was purified using 3 kDa centrifugal filter units (Amicon-Ultra-15 3K, Merck, Germany) and resuspended in 2.2 mM sodium citrate.

CeO_2_ NPs of 5 nm were synthesized by the chemical precipitation of cerium­(III) nitrate hexahydrate in a basic aqueous solution. Cerium­(III), 10 mM, was dissolved in 100 mL of Milli-Q water at room temperature. To this, 3 mL of a TMAOH solution (1 M) was added slowly at room temperature under vigorous stirring (final concentration of 10 mM), and the mixture was allowed to age under mild stirring overnight.

### CeO_2_ NP Conjugation to MSA

To prevent the aggregation of nanoparticles in the bloodstream and to avoid hypotonic shock, nanoceria was conjugated with albumin from mouse serum (MSA, Merck, Germany). This conjugation was performed in 10 mM phosphate buffer (PB) at 4 °C for 24 h prior to injection.

### Determination of ROS Scavenging Capacity of CeO_2_ NPs

The reactive oxygen species (ROS) scavenging capacity of CeO_2_ nanoparticles (NPs) was determined using the Amplex red hydrogen peroxide assay kit (A22188, Thermo Fisher Scientific) according to the manufacturer’s instructions. Briefly, 50 μg/mL CeO_2_ NPs were incubated with varying concentrations of H_2_O_2_ (1.5, 7.5, and 12.5 μM) for 5 min, 48 h, 72 h, and 168 h. Subsequently, the Amplex red reagent/HRP working solution was added, and the mixtures were incubated for 30 min at room temperature. Fluorescence was measured using a Varioskan LUX multimode microplate reader (Thermo Fisher Scientific) with excitation/emission wavelengths set at 540/590 nm. The Amplex red hydrogen peroxide assay kit can detect as little as 10 picomoles of hydrogen peroxide or 10 μU/mL of horseradish peroxidase activity in a 100 μL assay volume.

### CeO_2_ NP Characterization

#### Bacterial Endotoxin (LAL) Test

Both synthesis and purification of NPs were performed under sterile conditions and with nonpyrogenic material. To ensure safe NPs for animal administration, CeO_2_ NPs were tested for LPS levels at the Echevarne analysis laboratory (Barcelona).

#### Transmission Electron Microscopy (TEM)

CeO_2_ NPs were visualized using high-resolution transmission electron microscopy (HRES-TEM) (Tecnai F20 S/TEM). For sample preparation, 10 μL of the as-synthesized solution was drop-cast onto a carbon-coated 200 mesh copper grid and allowed to dry at room temperature for at least 24 h. The average size and distribution of the nanoparticles were measured using ImageJ Analysis software with at least 2000 particles counted for accuracy.

#### UV–visible Spectra

UV–visible spectra were acquired using a Cary 60 spectrophotometer (Agilent Technologies, USA) over the wavelength range 250–800 nm. Measurements were conducted in 1.5 mL plastic cuvettes.

#### Determination of Band Gap Energy

The optical band gap energy (EgE_gEg) of the cerium oxide (CeO_2_) nanoparticles was determined using the Tauc method, which relates the optical absorbance to photon energy. The absorbance spectra were recorded using a UV–vis spectrophotometer, and the band gap was calculated based on the Tauc equation:

$$\left(\alpha h\nu {)}^{\left(1/n\right)}=A(h\nu -E_{g}\right)$$ 1

where *h* is Planck’s constant, ν is the photon’s frequency, α is the absorption coefficient, *E*_g_ is the band gap, and *A* is a proportionality constant. The absorption coefficient (α) can be calculated using the well-known relation deduced from Beer–Lambert’s relation (α = 2.303A/d), where *d* is the path length of the quartz cuvette and *A* is the absorbance determined from the UV–visible spectrum.

The nature of the electronic transition was determined by selecting an appropriate exponent nnn, which depends on whether the transition is direct or indirect:

- Direct allowed transitions: *n* = 1/2
- Direct forbidden transitions: *n* = 3/2
- Indirect allowed transitions: *n* = 2
- Indirect forbidden transitions: *n* = 3.

Based on prior studies, CeO_2_ NPs exhibit both direct and indirect allowed transitions. The characteristic absorption peak of CeO_2_ at 283 nm corresponds to charge transfer transitions from O^2–^ (O 2p) to Ce^4+^ (Ce 4f).

#### Dynamic Light Scattering (DLS) and ζ-Potential

The hydrodynamic size and ζ-potential of the nanoparticles were measured by using a Malvern ZetaSizer Nano ZS (Malvern Instruments, UK) with a light source wavelength of 532 nm and a fixed scattering angle of 173°. Measurements were conducted in a 1 cm path length cell at 25 °C. Three independent measurements were performed for each sample.

### X-ray Diffraction

XRD diffraction experiments were performed on a Malvern Panalytical X’pert Pro diffractometer, Cu Kα X-rays of wavelength (λ) = 1.5406 Å. The patterns were collected in the angle region between 20 and 95°(2θ). The crystallite size of the sample was estimated using Scherrer’s formula:

crystallitesize(averageinÅ)=Kλ/(bcos⁡θ)

where *K* is the Scherrer constant (typically 0.94 for full width at half-maximum (fwhm) of spherical crystals with cubic symmetry), λ is the wavelength of the radiation, β represents the line broadening at half-maximum intensity (fwhm), after subtracting the instrumental line broadening, and θ is the Bragg angle (diffraction angle of the peak). The instrumental line broadening was recorded by using a silicon polycrystalline substrate.

### X-ray Photoelectron Spectroscopy (XPS)

X-ray photoelectron spectroscopy (XPS) was performed on a SPECS system equipped with a monochromatic Al source operating at 300W and a Phoibos 150 analyzer. The pass energy of the hemispherical analyzer was set at 20 eV, and the energy step of the high-resolution spectra was set at 0.05 eV. Binding energy (BE) values were referred to the C 1s peak at 285.0 eV. Data processing was performed with the CasaXPS software. Cerium 3d spectra were analyzed using six peaks for Ce4+ (V, V″, V‴, U, U″, and U‴), corresponding to three pairs of spin–orbit doublets, and four peaks (two doublets) for Ce3+ (V0, V′, U0, and U′), based on the peak positions reported by Mullins et al., where U and V refer to the 3d3/2 and 3d5/2 spin–orbit components, respectively. Samples were prepared by drop-casting the sample onto a clean silicon wafer.

### Mice

Mice are bred and maintained under pathogen-free conditions at the local animal facility (C3M, INSERM U1065, Nice, France). Experimental procedures were carried out in compliance with protocols approved by the local ethical and experimentation committee (SBEA, Nice, France, authorization nos. 28790–2020121715244498 and B0608820).

#### Plck-GAPDH Mice and Tumor Transplantation into NSG Mice

The Plck-GAPDH mouse generation and tumor transplantation are described in Mondragon et al.^48^

#### Cerium Oxide Nanoparticle (CeO_2_NP) Treatment

Two weeks after Plck-GAPDH lymphoma engraftment, NSG mice were treated with CeO_2_ NPs via IV injection of 80 μg CeO_2_NPs (1 mg/mL CeO_2_ NPs conjugated with 10 mg/mL mouse serum albumin in 10 mM phosphate buffer) or the vehicle (10 mM PB supplemented with 10 mg/mL MSA), every 2 weeks for a duration of 2 months.

#### Organ Distribution and Cerium Content Determination

Digestions were carried out by using an Ethos Easy (Milestone) advanced microwave digestion system. First, the samples were defrosted and mixed with a digestion solution consisting of one part concentrated nitric acid and two parts water. The digestion process was performed at 200 °C for 90 min. After digestion, elemental cerium in the tissues was analyzed using ICP-MS (7900 ICP-MS, Agilent) at the Chemical Analysis Service of UAB, Barcelona.

### Flow Cytometry and Antibodies for Murine Immune Cells

Antibodies used for detailed phenotyping or intracellular staining by flow cytometry of murine T and B are listed here and acquired from Miltenyi: CD3 APCcy7 (130–102–306), CD4 FITC (130–102–541); CD8 PEcy7 (130–119–123), B220 FITC (130–110–845), PD-1 PE (130–111–800), CXCR5 APC (130–103–113), ICOS-VB (130–100–639) or BD Pharmingen/CD19 PE (553786), CD95 VB (562633); INFgamma APC (554413), GL-7 APC (561529) or E-bioscience: perforin PE (12–9392–82), granzyme B PEcy7 (25–8898–82).

Staining with MitoTracker Green (Fisher Scientific; 150 nM) was performed according to the manufacturer’s instructions followed by surface Marking before FACS analysis.

For analysis of ROS content by FACS the CellROX green flow cytometry assay kit (Thermofisher) was used according to the manufacurer’s instructions.

For intracellular staining of granzyme B, perforin and IFNγ splenocytes were stimulated for 5 h in PMA (phorbol 12-myristate-13-acetate; Sigma, # P8139)/ionomycin (Sigma, # I0634) in the presence of Golgi-stop (BD Biosciences, #555029) and upon surface staining (anti-CD4 and anti-CD8) cells were fixed and permeabilized using the Cytofix/Cytoperm kit and protocol (BD Biosciences; #554714).

All stainings were detected by using a MACSQuant flow cytometer (Miltenyi Biotec, Paris, France). Analysis of the FACS data was performed using MACSquantify ver. 2.11 (Miltenyi) and FlowJo Software.

### Confocal Microscopy Imaging

Plck-GAPDH CD4+PD-1^high^ and CD4+PD-1^–^ tumor cells or WT CD4+ splenocytes were stained with MitoTracker Green at a concentration of 50 nM (Fisher Scientific) and imaged by confocal microscopy (Nikon A1R confocal, objective 60X, laser 488). All images were acquired with the same parameters for the 3 conditions to permit quantification of the MitoTracker signal (laser intensity = 3, signal amplification = 70); 330 cells per condition were quantified.

### Quantification and Statistical Analysis

Statistical analysis was conducted using Microsoft Excel 2013 and Prism software v6.0 (GraphPad Software, La Jolla, CA, USA). Results are indicated as means ± SD (standard deviation) in the figure legends unless otherwise noted. For statistical testing of significance, a student’s *t* test or one-way ANOVA was used, followed by the Tukey range test to assess the significance among pairs of conditions; p values are indicated in the figure legends. A p-value <0.05 was considered to indicate statistical significance. Mice's survival curves were evaluated using the log-rank test to determine significance. All flow cytometry data shown are representative of at least three repeated experiments. Gene set enrichment analysis was performed as described above. All RNaseq data and Affymetrix data generated or used in this study have been deposited at GEO and are publicly available as of the date of publication. Accession numbers are listed in the Methods section or the Supporting Information. Any additional information to reanalyze the data reported in this paper is available from the lead contact upon request.

### AITL Patient and Healthy Donor Tfh Cell Gene Expression Analysis for ROS Signature

See the Supporting Information. All human blood and tissues were obtained after informed consent, and approval was obtained by the ethical commission of the hospitals according to the Helsinki Declaration.

### In Vitro Treatment of mAITL T Cell Lymphoma Biopsies with CeO_2_NPs

AITL mice that developed T cell lymphoma were sacrificed, and splenic biopsies were homogenized to single-cell mixtures. 5E5 cells were seeded/24-well plates in culture medium (RPMI/10% FCS/1% Pen/strep supplemented with 50 nM of β-mercaptomethanol and mIL-7 (10 ng/mL). Cells were treated with CeO_2_NPs (in 10 mM PB supplemented with 10 mg/mL MSA) were added at 50 or 100 μg/mL, or with vehicle (10 mM PB supplemented with 10 mg/mL MSA). Three days after the start of treatment, FACS analysis was performed to determine the % of the CD4+PD-1^high^ cells as well as the % of CellRox CD4+ cells as described above.

### Toxicity Evaluation of CeO_2_ NPs In Vivo

Healthy NSG mice were treated with CeO_2_NPs by IV injection of 80 μg CeO_2_NPs (1 mg/mL CeO_2_NPs conjugated with 10 mg/mL MSA in 10 mM phosphate buffer) or with vehicle (10 mM PB supplemented with 10 mg/mL MSA) biweekly for 2 months (see Figure S4A). Mice were kept for 2 more months. During the duration of the experiment, the weight and behavior of mice were determined once per week. At sacrifice, the different organs (liver, spleen, intestines, kidney, lungs, and stomach) were investigated for signs of inflammation or other abnormalities.

## Supplementary Material



## Notes

**S**upplementary discussion on DLS measurements and supplementary figures: (Figure S1) thermogravimetric analysis (TGA) of CeO2 NPs; (Figure S2) XPS spectra of CeO_2_ NPs before and after conjugation to MSA; (Figure S3) human AITL malignant Tfh cells are strongly enriched in a ROS gene signature as compared to other healthy T cell subsets; (Figure S4) no toxicity was revealed in healthy NSG mice upon multiple CeO_2_ NP injections (PDF)

## Abbreviations

ROS: reactive oxygen species
AITL: angioimmunoblastic T cell lymphoma
CeO2 NP: cerium oxide nanoparticles
FACS: fluorescence activating cell sorting
OXPHOS: oxidative phosphorylation
Tfh: follicular helper T cells
ETC: electron transport chain
