# Supplementary material for: Scavenging Reactive Oxygen Species by Cerium Oxide Nanoparticles Prevents Death in a Peripheral T Cell Lymphoma Preclinical Mouse Model
Source: ACS Nano. 2025 May 9;19(19):18644–60. doi: 10.1021/acsnano.5c02860 (PMC12512179; doi:10.1021/acsnano.5c02860)
Supplement: Supplementary file 1 [file nn5c02860_si_001.pdf]

SUPPLEMENTARY INFORMATION

Scavenging Reactive Oxygen Species by Cerium Oxide Nanoparticles prevents death in a peripheral T cell lymphoma preclinical mouse model.

Adrien Krug[1,2,10], Lena M. Ernst[3,10], Rana Mhaidly[1,2,10], Joana Ramis[3], Muriel F. Gusta[3], Neus G. Bastus[3], Adriana Martinez-Turtos[1], Marie Tosolini[4], Léa Di Mascio[1,2], Gamze Tari[5], Laurent Boyer[1], Philippe Gaulard[5,6], François Lemonnier[5], Jean-Ehrland Ricci[1,2,9], Els Verhoeyen[1,2, 8,9,] and Victor Puntes[3,9,*]*

[1]*Université Côte d'Azur, INSERM, C3M, 06204 Nice, France*

[2]*Equipe labellisée Ligue Contre le Cancer, 06204 Nice, France*

[3]*Vall d'Hebron Research Institute (VHIR), Passeig Vall d'Hebron 119-129, 08035 Barcelona, Spain; Catalan Institute of Nanoscience and Nanotechnology (ICN2), CSIC and BIST, Campus UAB, Bellaterra, 08193 Barcelona, Spain; Institució Catalana de Recerca I Estudis Avançats (ICREA), 08010 Barcelona, Spain; Networking Research Centre for Bioengineering, Biomaterials, and Nanomedicine (CIBER-BBN), Instituto de Salud Carlos III, 28029 Madrid, Spain*

[4]*CRCT, Université de Toulouse, Inserm, CNRS, Université Toulouse III-Paul Sabatier, Centre de Recherches en Cancérologie de Toulouse, 31100 Toulouse, France*

[5]*Université Paris-Est Créteil; Institut Mondor de Recherche Biomédicale, INSERMU955; Unité hémopathies lymphoïdes, Hôpitaux Universitaires Henri Mondor, Assistance publique des Hôpitaux de Paris, F-94010 Créteil, France*

[6]*AP-HP, Groupe hospitalo-universitaire Chenevier Mondor, département de pathologie, F-94010 Créteil, France*

[7]*AP-HP, Groupe hospitalo-universitaire Chenevier Mondor, Service Unité Hémopathies Lymphoides, F-94010 Créteil, France*

[8]*CIRI, Université de Lyon; INSERM U1111; ENS de Lyon; University Lyon1; CNRS, UMR5308, 69007 Lyon, France*

[9]*Co-senior authors*

[10]*These authors contributed equally to this work in terms of experimental input, concept and critical data analysis.*

Correspondance: Els Verhoeyen; C3M-U1065, 151 Route St Antoine de Ginestière, 06200 Nice ; Tel. 003362104423; els.verhoeyen@unice.fr and Victor Franco Puntes: Vall Hebron Institute of Research, 119 Pg. Vall d Hebron, 08035 Barcelona; victor.puntes@vhir.org

DLS artefactual peaks detailed discussion. These artefactual peaks often appear in highly concentrated NP samples, needed to have enough signal coming from the sample, and consequent multiple scattering[1]. This point is important for all scientists working with such ultra-small and relatively transparent NPs samples.

The rationale goes like this (*this explanation has been added to the supplementary information*):

Cerium oxide has a density of 7.22 gr/cm3, so a particle with sizes of a few microns (6,000) nm should sediment relatively fast.

For small spherical particles in a fluid, the terminal velocity v_t is given by **Stokes' law**:

$$v_t = \frac{2}{9}\frac{(\rho_p - \rho_f)gr^2}{\mu}$$

Where:

- ρ_p = 7.22 g/cm^3 = **7220 kg/m³** (density of the particle)
- ρ_f = density of the fluid (for water, ρ_f1000 kg/m^3)
- g = **9.81 m/s²** (acceleration due to gravity)
- r = **6 µm/2 = 3 µm = 3 × 10^{-6} m** (radius of the particle)
- µ = dynamic viscosity of the fluid (for water, $\mu \approx 1.0\times10^{-3}$ Pa·s)

Thus, terminal velocity: 0.122 mm/s and time to descend 1 cm: 81.94 seconds (~1.37 minutes)

Properly stored NPs are stable for months and always show the same peaks. Signal intensity does not decrease either in DLS or UV-vis spectroscopy, where, as described by Lamber-Beer law, the intensity of the light extinction is directly proportional to the concentration of the nanoparticles.

Therefore, the peak cannot be made of CeO2 NPs, even if the aggregates packing is loose and the density of the aggregates is lower than for the single crystal.

It could be aggregates or any material with a similar density to water. However, filtering the sample through a 6,000 kDa (about 5 nm in diameter) does not separate these aggregates, having the sample before and after filtration, the retentate and the filtrate, the identical optical signatures in DLS and UV-vis spectroscopy.

Note also that the sample does not show any aggregation in the UVvis spectra, as aggregated and still soluble CeO2NPs display. These are for 100 nm aggregated CeO2 NPs sample:

[1] Jia, Z.; Li, J.; Gao, L.; Yang, D.; Kanaev, A. Dynamic Light Scattering: A Powerful Tool for In Situ Nanoparticle Sizing. *Colloids Interfaces* **2023**, *7*, 15. https://doi.org/10.3390/colloids7010015

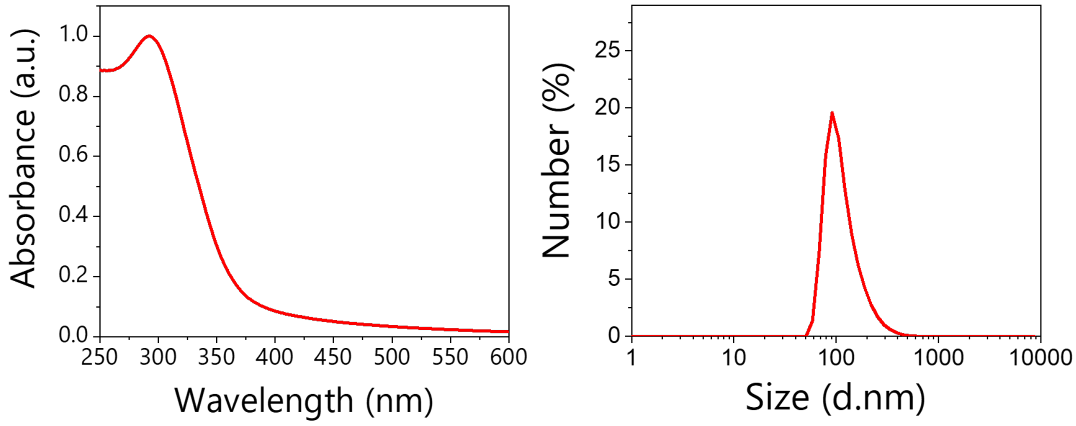

UVvis spectrum and DLS diameter size distribution of 100 nm aggregated CeO2 NPs.

Furthermore, centrifugation of the sample at 15.000 g does not change its optical signatures or intensity, further ruling out the presence of contaminants or aggregates.

Therefore, the signal coming from 6,000 nm is relatively weightless (no sedimentation despite size), *sizeless* (not filtered despite size), and transparent (no signal in the UV-vis spectra), and it is hard to imagine how this could correspond to CeO_2 aggregates.

Before injection, samples are filtered through a 220 nm (0.220 um) disinfection filter for secure sterility, and an aliquot is sent to ICPMS to confirm the dose (checking that the filter does not retain NPs).

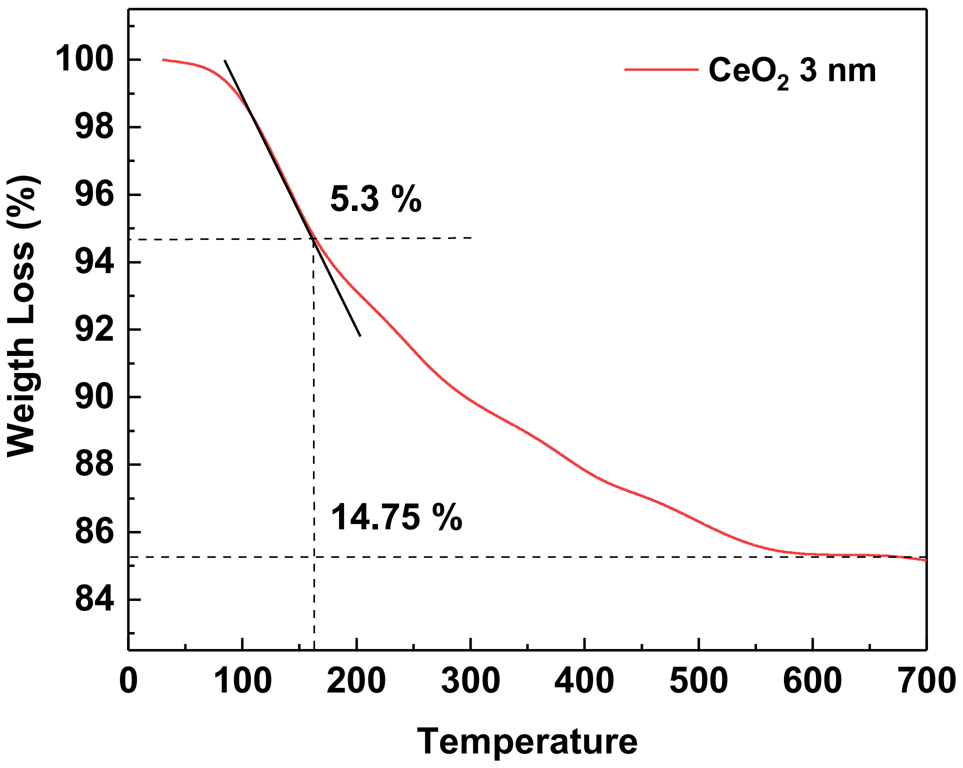

Supplementary Figure S1. Thermogravimetric analysis (TGA) of 3 nm CeO_2 nanoparticles. The thermogravimetric analysis was conducted under a nitrogen atmosphere with a 20.0 mL/min flow rate. The temperature was increased from 30 °C to 700 °C at a heating rate of 11.0 °C/min.

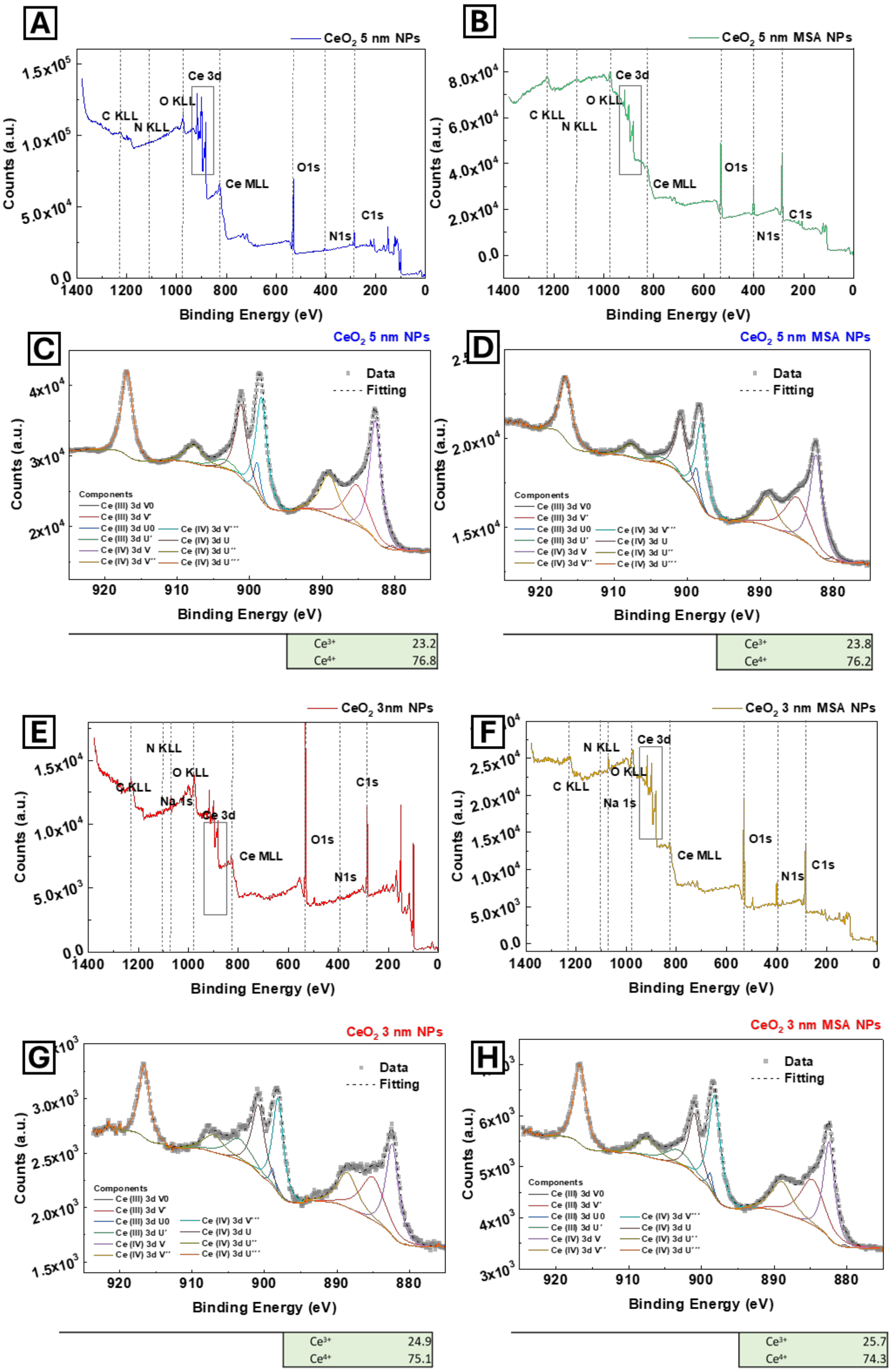

Supplementary Figure S2. XPS survey spectra of 5 nm and 3 nm CeO_2 NPs before (A: 5 nm, E: 3 nm) and after (B: 5 nm, F: 3 nm) conjugation with MSA. The wide-scan spectra exhibit characteristic peaks at approximately 285 eV, 400 eV, and 530 eV, corresponding to the C 1s, N 1s, and O 1s regions. **Ce 3d XPS** spectra of 5 nm and 3 nm CeO_2 NPs before (C: 5 nm, G: 3 nm) and after (D: 5 nm, H: 3 nm) MSA conjugation. Here, V and U denote the $3d_{5}/_{2}$ and $3d_{3}/_{2}$ spin-orbit levels, respectively.

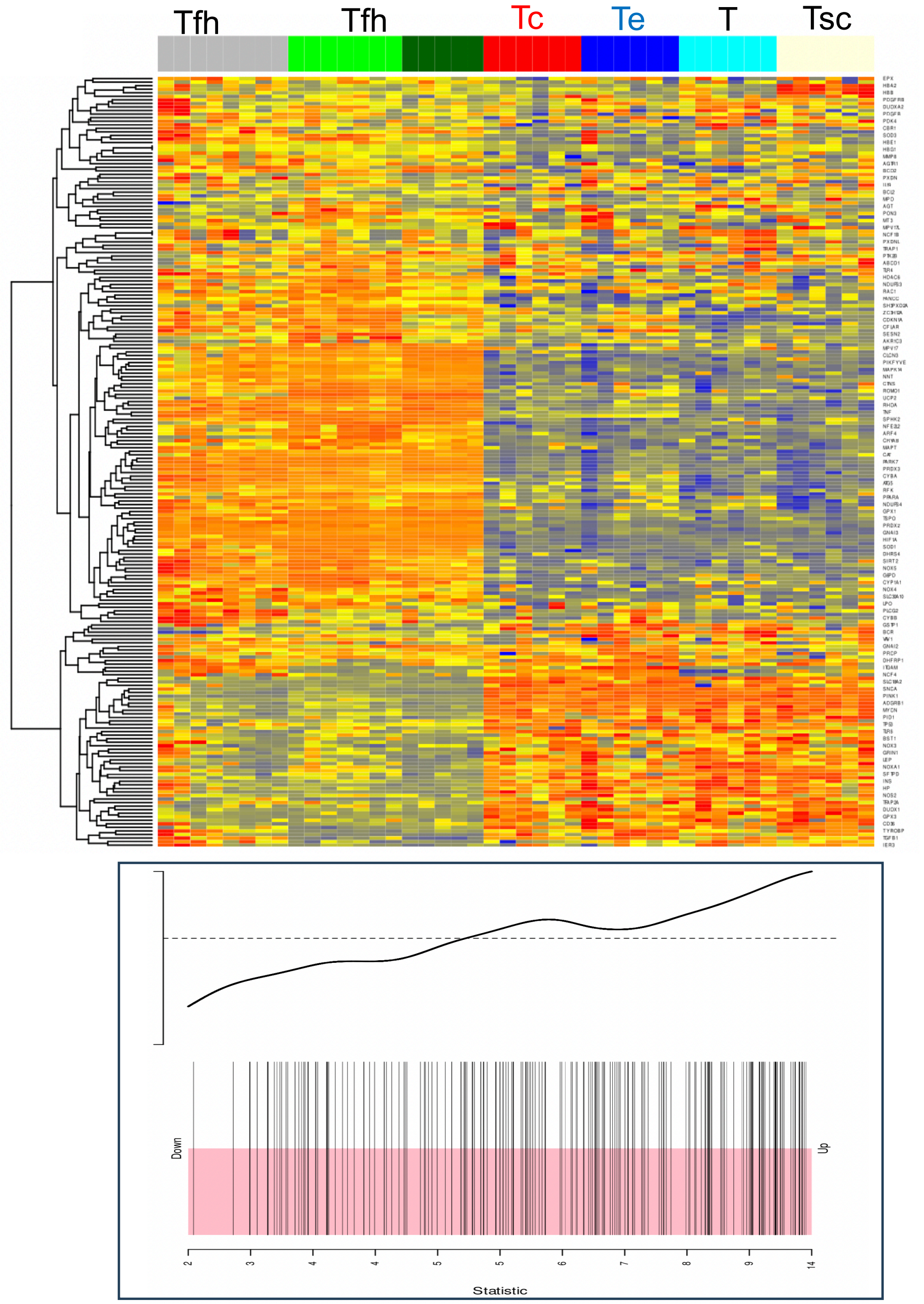

Supplementary Figure S3. Human AITL malignant Tfh cells are strongly enriched in a ROS gene signature compared to other healthy T cell subsets. (A) Heatmap for GSEA data of 231 genes implicated in ROS-related pathways, comparing GSEA data of isolated Tfh cells from AITL patients (n=8) versus healthy donor Tfh cells (n=12), central memory (Tcm, n=6), effector memory (Tem, n=6), naïve (Tn, n=6) and stem cell memory (Tsc, n=6) T cells. (B) The corresponding GSEA for the ROS signature genes in AITL Tfh is indicated. For all genes with enrichment score > 0 (black bars in the pink zone), expression is upregulated in hAITL. Kolmogrov-Smimov (KS) test.

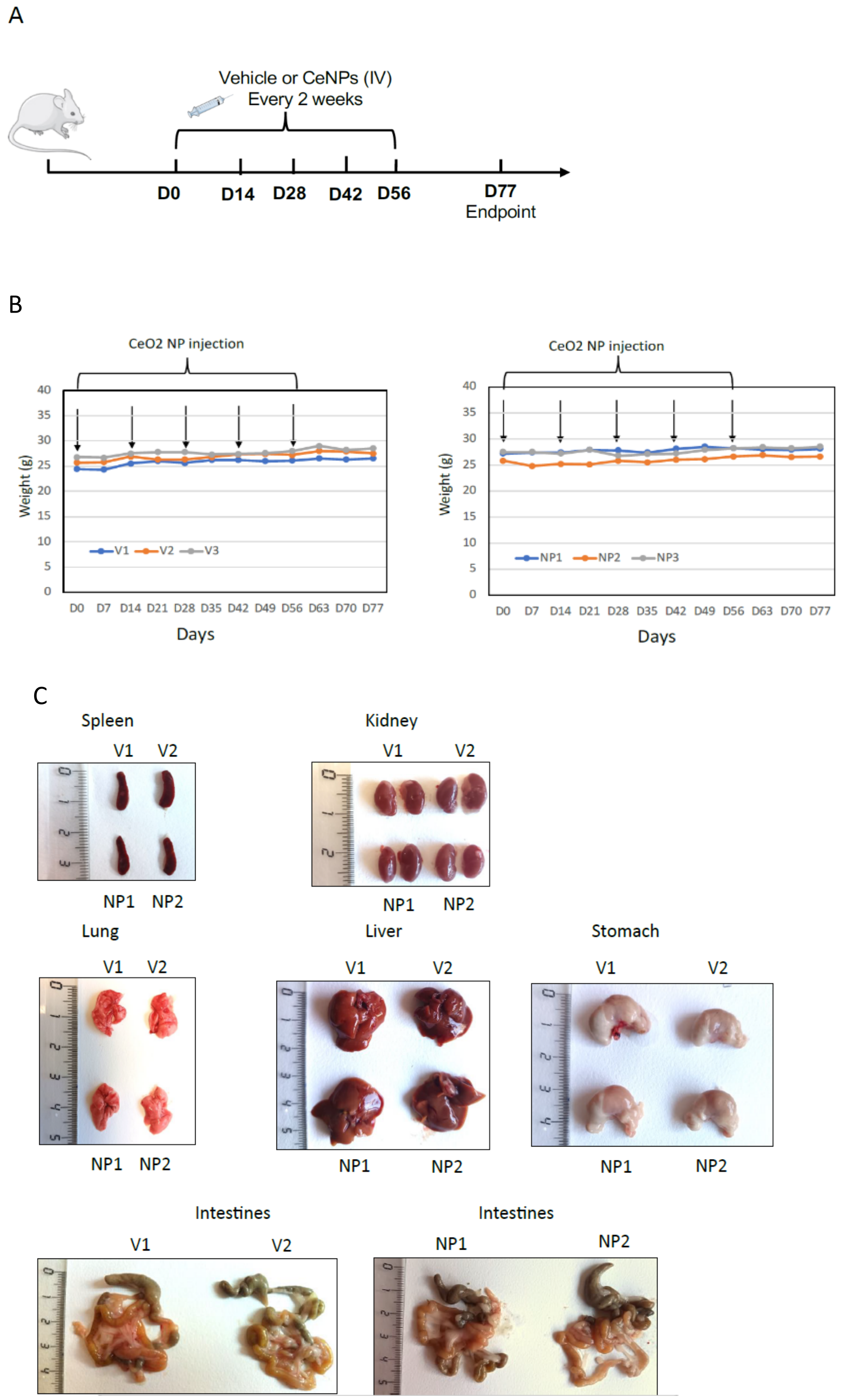

Supplementary Figure S4. No toxicity was revealed in healthy NSG mice upon multiple CeO_2 NP injections. (A) Schematic representation of the treatment with the NPs (n=3) or Vehicle (n=3) to evaluate toxicity. NSG mice were injected at days 0, 14, 28, 42 and 56 with CeO_2 NPs-MSA (80 µg NPs at 1mg/ml NPs in 10 mM Phosphate Buffer (PB) supplemented with 10mg/ml MSA) or vehicle (10 mM PB supplemented with 10mg/ml MSA). **(B)** The weight of the NSG mice injected with vehicle (V) or with CeO_2 NPs (NP) for the duration of the experiment shown in A. (**C**) Comparison of the different organs (spleen, kidney, lung, liver, stomach, and intestines) at sacrifice (day 77).
